# Hierarchical brain dynamics supporting visual perceptual transitions

**DOI:** 10.1126/sciadv.aea3919

**Published:** 2026-05-08

**Authors:** Max Levinson, Alice E. Waitt, Katharina Duecker, Syanah C. Wynn, Ole Jensen, Sylvain Baillet

**Affiliations:** ^1^ Montreal Neurological Institute, McGill University, Montreal, Quebec, Canada.; ^2^ Neuroscience Institute, New York University Grossman School of Medicine, New York, NY, USA.; ^3^ Centre for Human Brain Health, School of Psychology, University of Birmingham, Birmingham, UK.; ^4^ Department of Neuroscience, Brown University, Providence, RI, USA.; ^5^ Oxford Centre for Human Brain Activity, Oxford Centre for Integrative Neuroimaging, Department of Psychiatry, University of Oxford, Oxford, UK.; ^6^ Department of Experimental Psychology, University of Oxford, Oxford, UK.; ^7^ Centre de Recherche du Centre Hospitalier de l’Université de Montréal, Montréal, Quebec, Canada.; ^8^ Department of Neuroscience, Université de Montréal, Montréal, Québec, Canada.

## Abstract

A longstanding debate in consciousness research concerns whether subjective perceptual experiences arise primarily from activity in sensory cortices or rely critically on inferences made in higher-order brain regions. We address this question using a compelling visual illusion (perceptual filling-in) that isolates neural processes underlying transitions from veridical to illusory conscious experience. Using whole-brain magnetoencephalographic imaging and rapid invisible frequency tagging, we tracked cortical dynamics during filling-in and assessed their modulation by microsaccadic eye movements, which are known to delay the illusion. We found that transitions in conscious perception involved two dissociable mechanisms: (i) boundary fading in visual cortex, reflected by increased excitability and reduced alpha-band activity, consistent with a shift in excitation-inhibition balance, and (ii) higher-order perceptual monitoring processes involving motor cortex, indexed by decreased high-alpha and beta-band activity. Microsaccades selectively reset both processes. These findings support a hierarchical framework in which visual and motor systems jointly shape transitions in conscious visual experience.

## Teaser

Illusory transitions in conscious visual perception involve both early visual cortex and higher-order motor cortices.

## INTRODUCTION

Theories of conscious perception disagree on the neural origin of subjective experiences. A predominant question is whether conscious perception involves primarily sensory cortex activity driven by external stimuli or perceptual inferences generated within higher-order associative cortical regions (*1*–*3*). One phenomenon particularly suited to address this debate is illusory perceptual filling-in (*4*, *5*) (henceforth simply “filling-in”). To elicit filling-in, observers maintain gaze at a single fixation point while viewing a visual stimulus for several seconds. Eventually—for reasons that remain unclear—the boundary between two adjacent regions (e.g., a red spot on a blue background) appears to fade. The resulting phenomenology is of perceptual uniformity: The adjacent regions appear to blend and share identical features (e.g., forming a purple surface). The effect is observed across several experimental paradigms, including Troxler fading (*6*, *7*), artificial scotoma (*8*), and the uniformity illusion (*9*). Filling-in offers distinct advantages over other paradigms for studying the neural basis of conscious perception (*4*). Unlike masking or binocular rivalry, for example, filling-in permits direct observation and manipulation of the transition from veridical to nonveridical perception, thereby isolating the processes that actively construct subjective perceptual experience from those that may unconsciously represent stimulus properties.

The neural mechanisms underlying filling-in are poorly understood. A central question concerns the precise role of retinotopic visual cortices. Different studies across multiple modalities, from single-unit neuronal spiking to functional magnetic resonance imaging (fMRI), implicate different subregions of the early visual cortex (*10*–*13*) and even disagree on effect directions, with some results showing increased activation during filling-in (*10*, *12*, *14*) and others deactivation (*11*, *12*, *15*).

Surprisingly, none of these prior neurophysiological investigations leveraged a well-documented, natural perturbation of filling-in processes. Microsaccades—small, involuntary eye movements occurring one to three times per second—can prevent or delay the illusion (*16*–*20*). Microsaccades are thought to counteract reductions in excitatory neural activity, or adaptation, that accumulates during sustained gaze (*21*). Visual cortical neurons adapt during prolonged viewing of unchanging stimuli (*22*, *23*), and this adaptation likely contributes to illusory filling-in. By reversing adaptation and preventing a failure of boundary perception, microsaccades should directly interfere with the neural mechanisms underlying filling-in. Measuring their neural consequences can thereby help resolve uncertainty about which neural effects genuinely contribute to the filling-in experience.

Regarding the larger debate around neural mechanisms of conscious perception, it is unclear whether filling-in arises exclusively from retinotopically organized activity within visual cortex or involves higher-order inferential processes. Some authors propose that boundary adaptation triggers lateral spreading of excitation via horizontal connections in early visual areas (*24*, *25*). This hypothesized cortical phenomenon is a form of "isomorphic" filling-in—wherein percept-related neural activity propagates across retinotopic visual cortex, exactly mirroring the spread of visual information across the perceived visual field. Isomorphic filling-in could occur either automatically through intrinsic visual cortex dynamics or depend on a top-down initiator signal from higher-order cortical regions, as suggested by previous work (*12*, *26*, *27*). The present work aims to test this specific form of isomorphic filling-in via lateral spreading of bottom-up signals, but an isomorphism could also occur via feedback connections that do not alter feedforward visual signals.

Alternatively, "symbolic" filling-in entails a possible higher-order representation of perceptual content, outside of retinotopic visual cortex, without a direct isomorphism in visual cortex (*28*). There are therefore three competing possibilities for the neural hierarchy underlying filling-in after the boundary representation is lost: (i) automatic, low-level isomorphic filling-in alone (*24*, *25*); (ii) a top-down perceptual inference causes isomorphic filling-in (*12*, *26*); (iii) a higher-order inference instantiates the illusion via symbolic filling-in (*28*, *29*), perhaps informed by lower-level visual activity.

We predict that higher-order inference plays a role (hypotheses 2 or 3), but it is still unclear whether isomorphic filling-in truly occurs. To adjudicate between these three hypotheses, the present study is designed to detect feedforward isomorphic filling-in and/or higher-order inferential processing. Convincing evidence for isomorphic filling-in but no plausible higher-order process would support hypothesis 1 (low-level isomorphism). Evidence for both processes would support hypothesis 2 (top-down controlled isomorphism). Evidence for higher-order processing but no evidence for isomorphic filling-in would support hypothesis 3 (symbolic filling-in).

Previously, we proposed a visuomotor explanation for the emergence of filling-in (*4*). This hypothesis states that as visual input signals weaken in the absence of eye movements (*22*), motor cortical areas monitor the progressively diminishing perceptual evidence for stimulus boundaries through rhythmic activity in the alpha-beta frequency range (8 to 35 Hz). This builds on evidence that motor cortex contributes not only to movement but also to perceptual decision-making, subjective confidence judgments, and predictive inference (*30*–*33*). These findings position motor circuits as plausible “higher-order” contributors to the content of conscious perception (*30*, *32*, *34*), on top of controlling the motor output of perceptual decisions. Alpha/beta synchronization in motor cortices specifically has been associated with the tracking of sensory evidence related to ongoing perceptual experience (*31*, *33*, *35*, *36*). According to our hypothesis, higher-order mechanisms—including motor cortex within a broader decision-making network, likely taking inputs from related parietal and prefrontal regions—contribute to inference about visual field uniformity once bottom-up boundary evidence from visual cortex falls below a critical threshold. One goal of the present study is to determine whether the subsequent neural representation of the perceptually filled-in surface is isomorphic (hypothesis 2 above) or symbolic (hypothesis 3). Furthermore, a plausible framework based on this visuomotor hypothesis must account for how microsaccades counteract either neural component and thereby prevent perceptual filling-in.

The present study directly tested three core predictions of the visuomotor hypothesis for filling-in: (i) Bottom-up sensory signals weaken immediately before filling-in, reflecting boundary adaptation (*26*); (ii) filling-in follows alpha/beta power modulations in motor cortices (*28*–*34*); and (iii) microsaccades reverse both of these effects (*16*–*20*). We recorded source-imaged magnetoencephalography (MEG) (*37*) and high-resolution eye-tracking while participants observed filling-in with the uniformity illusion (*4*, *9*), in which isoluminant color appears to spread between a large central disk and the surrounding periphery. We used rapid invisible frequency tagging (RIFT) (*38**,* *39*) to continuously monitor excitability in visual cortical regions via imperceptible stimulus flicker (*40*, *41*). The stimulus center and periphery were tagged with different fast frequencies. This design allows for testing both low-level lateral interactions in visual cortex (isomorphic filling-in) and higher-order inferential mechanisms. We first predicted that the RIFT responses would diminish over time because of adaptation of neural excitation and that other neural proxies for excitation-inhibition balance, such as aperiodic activity (*42*–*44*) and occipital alpha power (*45*–*48*), would also reflect a decrease in cortical excitability. To assess isomorphic filling-in, we tested whether independent RIFT responses to central and peripheral flicker interacted with each other or spread across retinotopic cortex. To identify higher-order contributions to filling-in, we measured alpha-beta oscillatory power in motor cortex.

Our results support all three predictions of the visuomotor hypothesis while crucially clarifying their details. We found evidence that perceptual filling-in follows greater adaptation of inhibitory, relative to that of excitatory, signaling in visual cortex—with no evidence for subsequent isomorphic filling-in—and a decrease in alpha-beta oscillatory power in motor cortex. In line with the prior literature underlying the visuomotor hypothesis, we interpret this power decrease as a signature of higher-order inference: a cortical decision that the available sensory evidence no longer supports boundary segmentation. These findings are consistent with symbolic filling-in and provide a neurophysiological basis for hierarchical interactions that shape conscious perceptual experience.

## RESULTS

### Task and behavior

**Fig. 1.**
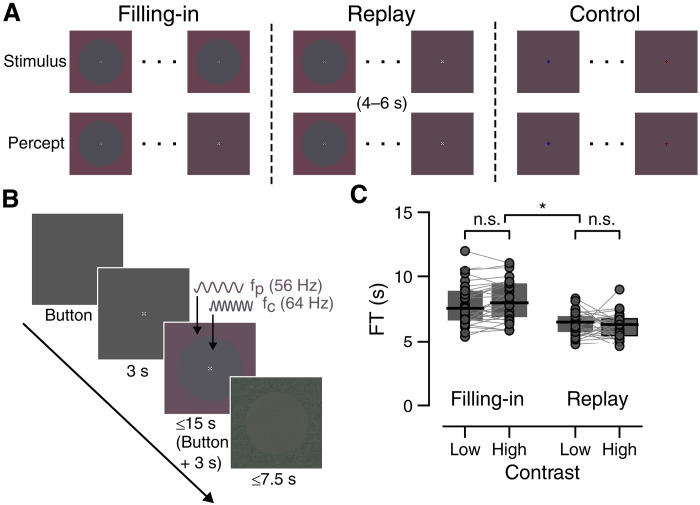
Task design and behavior. (**A**) Schematic illustration of the three task conditions. Left: Perceptual filling-in trials. After prolonged fixation, the colors of the central disk and surrounding periphery appear to perceptually blend into a uniform surface. Middle: Replay catch trials. The stimulus physically transitions into a uniform color, mimicking the filling-in illusion. The replay transition begins randomly between 4 and 6 s after stimulus onset and lasts 3 s. Right: Control trials. The stimulus is uniform from the outset, and participants instead report color changes of the central fixation cross. (**B**) Trial sequence diagram. Participants initiated each trial with a button press. After an initial 3-s fixation period, the stimulus appeared and remained visible until 3 s after the participants reported perceptual filling-in (by pressing the button again), or for a maximum duration of 15 s. The central disk flickered at 64 Hz and the surrounding periphery at 56 Hz, using RIFT. Each trial ended with dynamic noise overlaid on a color-inverted version of the stimulus to mitigate retinal afterimages. This noise stimulus was presented for half the duration of the filling-in stimulus presentation. (**C**) Average FT for each trial type, separated by two stimulus color contrast levels. There was no significant difference between contrasts in either filling-in (left, *P* = 0.094) or replay (right, *P* = 0.46) trials (two-sided Wilcoxon signed-rank tests). Replay trials were significantly shorter than filling-in trials (*P = 2.56 × 10^−6^, two-sided Wilcoxon signed-rank test). Each disk represents an individual participant. Boxplots indicate median and quartiles. n.s., not significant.

The sequences of perceptual experience during filling-in, replay, and control trials are illustrated in Fig. 1A. In the main task (Fig. 1A, left, and Fig. 1B), two isoluminant colors in a central disk (gray) and the surrounding periphery (purple) appeared to merge across their boundary (*4*, *9*). Half of the trials contained low color contrast stimuli, and half contained high color contrast stimuli (see Materials and Methods, Stimuli and behavioral tasks). Participants were instructed to maintain central fixation throughout each trial and press a button with their right index finger when they perceived the two regions of the stimulus to fully merge into a single, uniformly colored image (purple-gray; Fig. 1A, left). This single report of perceptual uniformity was the only possible response the participants could provide. To continuously track neural responses to the two stimulus regions throughout each trial (see Materials and Methods, RIFT presentation), the central disk flickered at 64 Hz and the periphery at 56 Hz.

Participants reported experiencing filling-in in 86 ± 2.81% (mean ± SEM) of filling-in trials. We defined filling-in time (FT) as the duration of central fixation required before experiencing filling-in, measured between stimulus onset and the button press. The average FT was 8.01 ± 0.29 s, replicating our previous observations from two independent samples of participants (*4*, *49*). FT did not significantly differ between low and high color contrast stimuli (7.86 ± 0.3 s versus 8.15 ± 0.29 s, respectively; Wilcoxon signed-rank test, *W* = 140, *P* = 0.094; Fig. 1C, left). Consequently, trials from both contrast conditions were combined for all subsequent analyses.

To monitor task compliance, we interleaved 12 “replay” catch trials in which the stimulus physically simulated the filling-in experience by blending the center and periphery colors on-screen (Fig. 1A, middle). Although illusory filling-in occurs quickly, an identically fast reduction in stimulus color contrast would produce salient, nonuniform afterimages (*50*, *51*). The replay effect therefore occurred gradually over 3 s to ensure that the participants would experience color uniformity at some point within this window. Color mixing began between 4 and 6 s after trial onset, earlier than the expected illusory FT based on previous studies (*4*, *49*) to reduce the chance that illusory filling-in would occur before the physical replay transition. We expected participants to report filling-in shortly after this replay event began. As anticipated, participants reported filling-in in almost all (94 ± 2.51%) of the replay trials, and the FT was significantly shorter in the replay condition than in illusory filling-in trials (mean replay FT = 6.31 ± 0.13 s; Wilcoxon signed-rank test, *W* = 0, *P* = 2.56 × 10^−6^; Fig. 1C). All participants exhibited this pattern, confirming that they followed task instructions. Because replay FTs were triggered by actual stimulus changes, they were more densely clustered around the mean than illusion trial FTs (fig. S1). No significant difference was observed between the two replay color contrasts (6.37 ± 0.17 s versus 6.25 ± 0.18 s; Wilcoxon signed-rank test, *W* = 183, *P* = 0.46; Fig. 1C, right). Replay trials were not analyzed further.

In the control condition—the final task block—participants viewed a uniformly colored stimulus and were instructed to press a button whenever the central fixation cross changed color between red and blue. Each trial contained multiple color changes occurring at mostly random times, but we only analyzed responses to the final color change in each trial, which always occurred at a fixed time point (6.5 s). This time was chosen to match the expected mean FT in the main task, based on previous studies using similar stimuli (*4*, *49*). The average reaction time for this response was 0.39 ± 0.014 s, or 6.89 s since stimulus onset. The purpose of this control condition was to measure neural activity elicited by a similarly uniform visual experience, identical RIFT stimulation, and button presses without the conscious experience of perceptual filling-in.

### RIFT signal amplitudes increase before filling-in reports

**Fig. 2.**
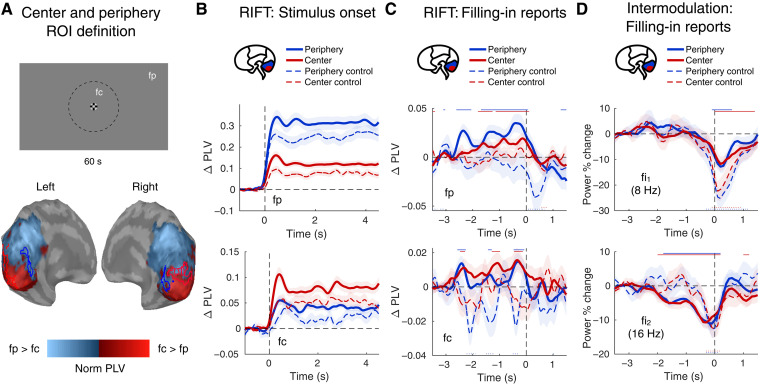
RIFT neurophysiological responses. (**A**) Definition of center and periphery ROIs using a localizer task. Top: A central circular region flickered sinusoidally at 64 Hz (fc), and the surrounding peripheral region flickered at 56 Hz (fp). These stimulus regions and flicker frequencies are identical to those used in the main task, but both regions were filled with the same gray color. The dashed circle indicating the boundary between center and periphery flicker regions was not visible to participants and is shown here only for illustration purposes. Bottom: Group-averaged cortical responses showing preferential responses to fc (red) or fp (blue). The plot displays the normalized difference in phase-locking values (PLV) between fc and fp (fc minus fp) for occipital sources only. Examples of individual ROIs from one participant are outlined. (**B**) RIFT cortical responses aligned to stimulus onset in the filling-in and control tasks. The top panels depict phase-locking to the peripheral stimulus frequency, which is higher in representations of peripheral (blue) compared with central (red) vision. The bottom panels depict phase-locking to the central stimulus frequency, which shows the opposite pattern. Solid lines indicate filling-in trial data; dashed lines show control trial data. (**C**) RIFT cortical responses aligned to the button-press indicating the subjective report of filling-in. (**D**) Intermodulation frequency components (fi_1_, 8 Hz and fi_2_, 16 Hz) aligned to filling-in reports. In all plots, statistical significance (*P* < 0.01, cluster-based permutation test) is indicated by horizontal colored lines at the top (filling-in task) or dotted lines at the bottom (control task) of each panel. Line colors correspond to the respective ROIs. Data in (C) and (D) are plotted relative to a baseline period (−3.5 to −3 s before report).

In an independent localizer run we identified two cortical locations in each participant that responded most strongly to rapid visual flicker in the stimulus center (fc, 64 Hz) or in the periphery (fp, 56 Hz). These regions corresponded to the posterior occipital pole and the more anterior aspect of the occipital cortex, respectively (Fig. 2A), as expected from retinotopic organization (*52*). We defined the RIFT response as the time-resolved phase-locking value (PLVt) between MEG activity and the stimulus flicker time course, as measured with a photodiode. In the filling-in condition, RIFT responses in both regions were stable after stimulus onset. The center and periphery maintained their frequency specificity throughout the trial duration (Fig. 2B).

Just before participants’ reports of filling-in, we observed a significant increase in RIFT responses relative to an earlier baseline period within the same trial (cluster-based permutation test, *P* < 0.01; Fig. 2C). RIFT responses increased at both flicker frequencies and in both the central and peripheral regions. This enhancement was observed only during perceptual filling-in and not in control trials. Rather than reflecting typical adaptation-related suppression (*23*, *53*), these findings suggest that filling-in is unexpectedly accompanied by a widespread elevation in excitability across visual cortex. We hypothesized that this could be attributed to the overadaptation of inhibitory connections relative to excitatory connections.

### No evidence for isomorphic filling-in

Heightened excitability in visual cortex could potentially lead to isomorphic filling-in. If bottom-up excitation propagated across retinotopic cortex, then the RIFT response would subsequently spread and interact accordingly, leading to the following predictions: (i) RIFT responses would selectively increase in regions adjacent to their original stimulation areas—for instance, the periphery would gradually become more responsive to fc than to fp—and (ii) cortical responses at intermodulation frequencies (multiples of the difference between fc and fp) would increase until filling-in report. Intermodulation results from nonlinear interactions between two oscillations (*54*) and has been shown to increase when two sensory processes tagged with rapid frequencies interact, such as during multisensory integration (*55*). We hypothesized that an intermodulation signal might manifest if the two flicker frequencies laterally interact during filling-in.

The above observation that all RIFT responses increased in both center and periphery (Fig. 2C), rather than selectively spreading the central frequency into the peripheral region and vice versa, does not support this prediction of lateral spreading. To further rule out isomorphic filling-in, we next measured intermodulation at fi_1_ (the first intermodulation frequency, 8 Hz) and fi_2_ (16 Hz) to replicate reports of fi_1_ responses from previous frequency tagging studies of conscious perception (*14*, *56*). We did not observe a significant increase in intermodulation activity power (cluster-based permutation test, *P* > 0.01; Fig. 2D) nor consistent significant phase-locking at fi_1_ or fi_2_ (cluster-based permutation test, *P* > 0.01; fig. S2) during filling-in. Instead, we observed that power at both intermodulation frequencies decreased (cluster-based permutation test, *P* < 0.01; Fig. 2D). Note that it is possible that the two signals are processed in separate neuronal networks so that even if they do spread laterally, they may not produce intermodulation components. However, the absence of both predicted effects—selective spreading of RIFT responses and heightened intermodulation—suggests that perceptual filling-in does not result from an isomorphism in visual cortex but instead may require higher-order, symbolic processes.

### Excitation-inhibition balance in visual cortex before filling-in

We next aimed to further characterize the circuit-level changes in visual processing that may inform putative higher-order contributors to filling-in. On the basis of the observation of an increase in cortical excitability in the previous RIFT analysis, we hypothesized that perceptual filling-in is caused by an increase in excitation/inhibition (E/I) ratio across visual cortex, leading to unstable boundary representations. We therefore predicted that two indirect neurophysiological markers of cortical inhibition in the visual cortex would decrease before filling-in reports: the exponent and offset of the aperiodic components of the power spectrum (*42*–*44*) (see Materials and Methods, Time-resolved estimation of aperiodic spectral power), and alpha-band power (*45*–*48*). The exponent reflects inhibition relative to excitation (*44*, *57*, *58*), whereas the offset indexes broadband power linked to overall spiking activity (*43*, *59*, *60*). These measures are indirect markers of neural excitation-inhibition balance. Both of these measures reliably track cortical inhibition, although neither is a pure linear readout and they can also be influenced by other neuronal processes (*61*, *62*).

**Fig. 3.**
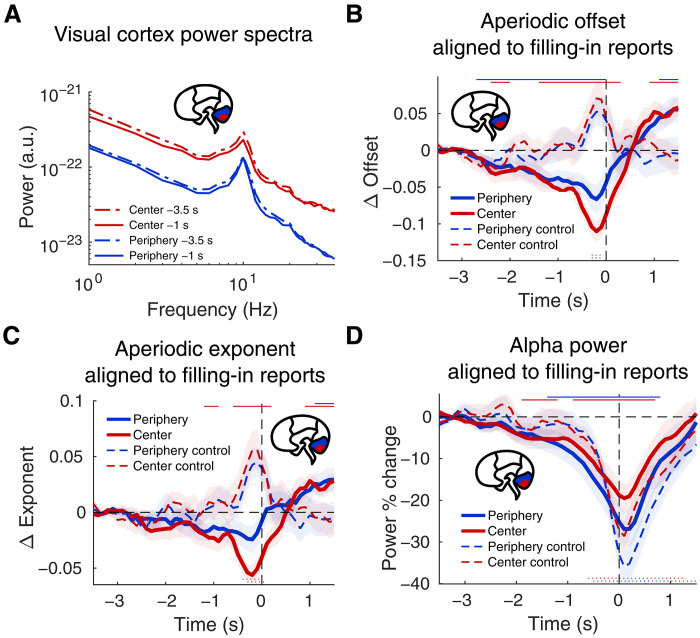
Spectral markers of E/I balance in visual cortex. (**A**) Average power spectral densities for central (red) and peripheral (blue) cortical ROIs at two time points: approximately 3.5 s (dashed lines) and 1 s (solid lines) before participants’ filling-in reports. A clear decrease in the aperiodic offset (broadband power level) is observed in both central and peripheral cortical ROIs over this time interval. (**B**) Aperiodic offset parameter aligned to the button press reporting the filling-in experience. Aperiodic offset significantly decreased in central (red) and peripheral (blue) cortical ROIs before filling-in. In contrast, the offset showed a brief but significant increase immediately before button presses in control trials. (**C**) Aperiodic exponent parameters aligned to the button press reporting the filling-in experience. Similar to (B), the aperiodic exponent also significantly decreased in central (red) and peripheral (blue) cortical ROIs before filling-in but not before button presses in control trials. (**D**) Aperiodic-corrected alpha-band (8 to 12 Hz) oscillatory power aligned to the button press. Alpha power significantly decreased in central (red) and peripheral (blue) cortical ROIs several seconds before a filling-in report. In contrast, alpha power did not decrease until just before button presses in control trials. In (B) to (D), statistical significance (*P* < 0.01, cluster-based permutation test) is indicated by solid lines at the top (filling-in task) or dotted lines at the bottom (control task), with colors corresponding to cortical ROIs indicated in the figure legend. Data in (B) to (D) are plotted relative to a baseline period (−3.5 to −3 s before report). a.u., arbitrary units.

Figure 3A shows the power spectra of neural activity in center and periphery regions of interest (ROIs), measured 3 and 0.5 s before filling-in reports. Using aperiodic parametrization of the power spectra, we found—consistent with our hypothesis—that both the exponent and offset of the aperiodic signal component decreased as the perceptual transition approached (cluster-based permutation test, *P* < 0.01; Fig. 3, B and C). The decrease in offset, or broadband power, suggests a reduction in overall spiking activity (both excitatory and inhibitory) (*43*, *59*, *60*), whereas the decrease in exponent indicates a relative reduction in inhibition compared with excitation (*44*, *57*, *58*). These decreases persisted for approximately 2 s before the button press and coincided temporally with the previously observed increase in RIFT responses. The effect was spatially confined to the visual cortex ROIs; no corresponding effect was observed in motor cortex ROIs (fig. S3), which instead showed a marked increase in aperiodic parameters beginning roughly 400 ms before the button press, which is consistent with movement execution rather than perceptual processing. Furthermore, both exponent and offset in visual cortex remained stable for several seconds after stimulus onset (fig. S4A), only declining closer to when the filling-in experience occurred.

Subsequently, we subtracted the aperiodic component from the power spectra at each time point to elucidate rhythmic low-frequency activity. We found that aperiodic-corrected alpha-band (8 to 12 Hz) activity in the occipital cortex also significantly declined before filling-in reports (Fig. 3D) over a similar time window as the aperiodic effect.

### Decreased alpha-beta activity in motor cortex before filling-in

**Fig. 4.**
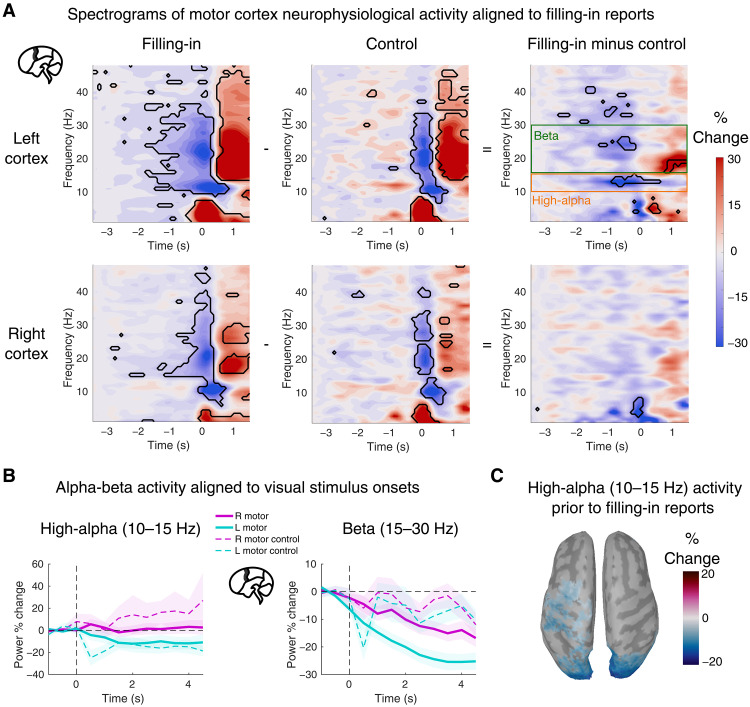
Rhythmic activity in motor cortex. (**A**) Time-frequency power changes in motor cortex aligned to the button press indicating a filling-in report (left column, *n* = 3852 trials) or a control task response (middle column, *n* = 700 trials). Statistically significant time-frequency clusters (*P* < 0.01, FDR-corrected permutation test) are outlined in black. The rightmost column shows the differential power (filling-in minus control task). (**B**) Motor-cortex rhythmic power aligned to stimulus onset, averaged within two frequency bands of interest defined from (A): high-alpha (10 to 15 Hz) and beta (15 to 30 Hz). Here, the beta frequency range was defined as the remainder of the canonical beta band (13 to 30 Hz) that was not included in the data-driven high-alpha band. This panel is for visualization purposes only; no statistical tests were performed. (**C**) Dorsal cortical view of whole-brain, baseline-corrected high-alpha-band (10 to 15 Hz) power, averaged from −1.25 to −0.75 s before the filling-in report. Colored regions indicate significant cortical activity (permutation test, uncorrected *P* < 0.05, minimum cluster size: 30 contiguous vertices).

We observed a significant reduction of alpha- and beta-band activity in the motor cortex, starting 1 s before the filling-in report [*P* < 0.01, false discovery rate (FDR)–corrected permutation test against baseline; Fig. 4A, left column]. Notably, the decrease of beta-band activity was bilateral, whereas the decrease in alpha-band activity was restricted to the left motor cortex, contralateral to the right index finger used for reporting filling-in. In uniform control trials, a similar decrease was observed from 400 ms before the button press (Fig. 4A, middle column), which may be attributed to movement preparation, and there was no obvious difference between the left and right hemispheres. Signal power was significantly weaker for filling-in versus control trials between 10 and 15 Hz and only in the left motor cortex (*P* < 0.01, FDR-corrected permutation test; Fig. 4A, rightmost column). Consequently, we focus subsequent motor-cortex alpha-band analyses on the higher frequency range of 10 to 15 Hz, termed “high-alpha”. Motor-cortex analyses in the beta band are restricted to 15 to 30 Hz.

On the basis of the visuomotor hypothesis, we predicted that if the left motor cortex continuously tracks the decay of bottom-up visual boundary evidence—starting strongly at stimulus onset and weakening over time—its alpha and beta activity should progressively decline throughout the trial, commencing immediately after stimulus onset. However, we observed that high-alpha activity in the motor cortex maintained a substantial level of stability beyond the initial second after stimulus onset (Fig. 4B, left). Conversely, beta-band activity exhibited a consistent, bilateral decline commencing at stimulus onset (Fig. 4B, right). Notably, this kind of gradual suppression was absent from the visual cortex at any frequency band (fig. S4B).

Given the apparent similarity in alpha-band activity time courses across motor and visual cortices, we assessed whether one underlying cortical source could have produced both effects via spatial leakage or cross-talk in MEG source imaging. We examined whole-brain high-alpha activity around the time window when the lateralized motor cortex effect emerged (−1.25 to −0.75 s before filling-in; Fig. 4A, left column). The resulting cortical surface distribution (Fig. 4C; uncorrected permutation test, *P* < 0.05) revealed two spatially distinct regions of significant high-alpha power activity. In addition, the visual cortex effect was bilateral, whereas the motor cortex effect was lateralized. This evidence against a common source supports our hypothesis of separate visual and motor cortical contributions to perceptual filling-in.

### Microsaccades restore both visual inhibition and motor high-alpha activity

**Fig. 5.**
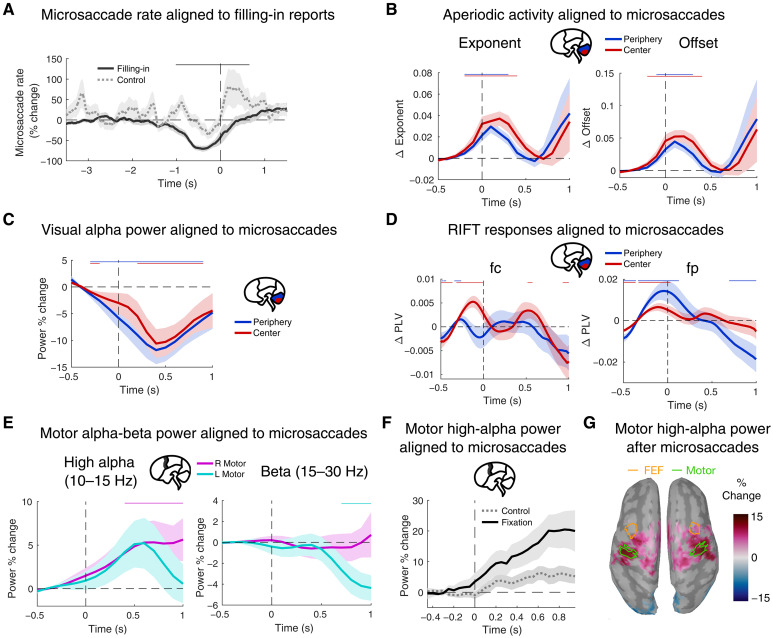
Neurophysiological changes in visual and motor cortical responses to microsaccades. *n* = 27 of 29 participants. (**A**) Average microsaccade rates aligned to the button press reporting perceptual filling-in. Microsaccade rates significantly decreased before filling-in reports (*n* = 3852 trials) but not before control responses (*n* = 700 trials). The thin black line at the top denotes a significant temporal cluster (cluster-based permutation test comparing filling-in versus control trials; *P* < 0.01). (**B**) Aperiodic exponent and offset parameters in visual cortex, aligned to microsaccade onsets. (**C**) Aperiodic-corrected alpha-band (8 to 12 Hz) oscillatory power in visual cortex, aligned to microsaccade onsets. (**D**) RIFT cortical responses aligned to microsaccade onsets. (**E**) Aperiodic-corrected high-alpha (10 to 15 Hz) and beta (15 to 30 Hz) power in motor cortex, aligned to microsaccade onsets. (**F**) Motor cortex high-alpha power aligned to microsaccade onsets and averaged across hemispheres. Microsaccade epochs were extracted from either control trials (gray dotted line) or from the fixation period before stimulus onset in filling-in trials (black solid line). High-alpha activity increases following microsaccades are clearly visible. This panel is for visualization only; no statistical tests were applied. (**G**) Dorsal cortical view of baseline-corrected high-alpha-band (10 to 15 Hz) power, averaged over the interval 500 to 750 ms following microsaccade onset. Colored regions indicate significant cortical activity (uncorrected permutation test, *P* < 0.05; minimum cluster size: 30 contiguous cortical vertices). Outlined regions indicate estimated locations of the FEF (orange) (*67*) and motor cortex ROIs (green; vertices included in >50% of individual participant ROIs). Unless otherwise specified, statistical significance from a cluster-based permutation test against baseline (*P* < 0.01) is indicated by horizontal colored lines at the top of each panel, with colors corresponding to the figure legend.

We characterized the associations between fixational microsaccades, brain activity, and behavioral reports of filling-in. We replicated the observation (*19*) of decreased microsaccade rates before filling-in reports compared to control button presses (Fig. 5A; cluster-based permutation test, *P* < 0.01). We then investigated the neurophysiology underlying this observation, with the expectation that microsaccades would counteract our previously observed neural correlates of filling-in.

In the visual cortex, microsaccades were associated with increased aperiodic exponent and offset (*P* < 0.01, cluster-based permutation test; Fig. 5B), compatible with enhanced inhibition relative to excitation. This finding may reflect the event-related neural response to microsaccades: a slow activity wave following changes in the retinal image (*63*). The cellular physiology of these event-related responses is not fully understood but is known to involve dynamic shifts of the E/I ratio (*64*). Nonetheless, we note that the increase in aperiodic parameters surrounding microsaccades contrasts with their decrease before filling-in reports, suggesting that microsaccades causally counteract a neural mechanism that may contribute to filling-in.

In contrast, we did not observe a decrease in RIFT response or an increase of occipital alpha activity immediately around microsaccades. Instead, alpha activity decreased with a delay (*P* < 0.01, cluster-based permutation test; Fig. 5C), and RIFT activity increased (Fig. 5D), reminiscent of the changes observed before filling-in reports rather than counteracting them.

In motor cortex, high alpha-band but not beta-band activity increased approximately 600 ms after the onset of microsaccades (*P* < 0.01, cluster-based permutation test; Fig. 5E). This increase contrasts with the decrease of high-alpha activity observed before filling-in reports (Fig. 4A). Notably, unlike the lateralized decrease associated with filling-in, this increase appeared to manifest bilaterally with a significant cluster in the right hemisphere (Fig. 5E). Furthermore, it was not specific to the filling-in condition but was also present in uniformly colored control trials and during the prestimulus fixation period at the onset of filling-in trials (Fig. 5F).

To determine whether the microsaccade-related increase in high-alpha activity (Fig. 5, E and F) was confined to motor cortex, we conducted a whole-brain analysis (FDR-corrected permutation test, *q* = 0.01). The delayed enhancement of high-alpha power, emerging 500 to 750 ms after microsaccade onset, was localized bilaterally to the precentral gyrus (Fig. 5G), suggesting that the effect is present in both hemispheres. We also examined whether this activity might originate from the frontal eye fields (FEF), a key oculomotor region implicated in saccade generation (*65*) and top-down modulation of visual processing (*66*). In Fig. 5G, the putative location of the human FEF (*67*) is outlined in orange. In contrast, green outlines in Fig. 5G denote our functionally defined motor cortex ROI—comprising all vertices that appeared in the motor cortex ROIs of at least 50% of the participants. The overlap with high-alpha activity indicates that the late increase in high-alpha power following microsaccades originates in precentral motor cortex rather than the FEFs. This result demonstrates a direct impact of microsaccades on a higher-order neural process in motor cortex that is associated with perceptual filling-in, independent of oculomotor generators in FEF.

### No confound from microsaccade rate decrease

Because a decrease in microsaccade rate typically accompanies filling-in, there is a potential confound: Our observed neurophysiological effects might not be related to filling-in at all but instead only reflect reduced eye movements. This concern was previously raised by Hsieh and Tse (*11*) as a possible alternative explanation for visual cortical effects. To address this, we isolated a subset of trials (26%) that exhibited no detectable microsaccades during the analysis window (up to −3.5 s before button press). In these trials, the absence of microsaccade production rules out eye movement-related changes as a source of neural modulation, allowing any observed effects to be more confidently attributed to filling-in.

Analyses restricted to these microsaccade-free trials reproduced our key findings: (i) increased RIFT responses in occipital cortex (fig. S5A), (ii) decreased aperiodic spectral parameters in occipital regions (fig. S5B), and (iii) reduced alpha-beta activity in motor cortices (fig. S5, D and E). In this subset, we did not observe the same sustained alpha-band modulation in visual cortex as in the full dataset. Instead, we found a brief increase in alpha power approximately 2.5 s before filling-in, followed by a decline below baseline levels (see fig. S5C versus Fig. 3D). However, this alpha-band effect remained weaker in magnitude than that observed in control trials (fig. S5C, right).

Collectively, these findings indicate that the neurophysiological correlates of filling-in reports are likely not solely attributable to sporadic reductions in the microsaccade rate because they still occur in the absence of any detectable microsaccades. Moreover, by reproducing our primary findings using only 26% of the filling-in trials, we demonstrate that the reported differences between filling-in and control trials were not attributable to a higher number of trials in the filling-in condition.

## DISCUSSION

Here, we provide converging evidence from source-imaged MEG and fixational eye movements for a two-part, hierarchical brain mechanism governing perceptual filling-in—spanning from lower-order visual cortex to higher-order motor cortex. Three complementary observations with coordinated timing suggest that perceived boundary fading before filling-in is associated with a shift in excitation-inhibition balance within visual cortex. Specifically, we observed increased RIFT phase-locking, decreased aperiodic exponent and offset, and decreased alpha-band activity preceding reports. These findings indicate that, although prolonged gaze fixation causes neuronal adaptation, boundary fading unexpectedly does not arise from decay of visually evoked excitation. Instead, they are most consistent with an account in which inhibition diminishes relative to excitation, facilitating a transition in perceptual experience.

Simultaneously, in the motor cortex, we observed a reduction in both high-alpha (10 to 15 Hz) and beta-band (15 to 30 Hz) periodic activity. We propose that these dynamics are consistent with the monitoring of perceptual evidence for a boundary as part of a higher-order perceptual decision-making process. In contrast, microsaccades were associated with increased aperiodic activity in the visual cortex and elevated high-alpha power in motor cortex—both of which appear to counteract the neural correlates of filling-in. These effects provide a plausible neural explanation for why microsaccades reliably delay the emergence of the filling-in percept.

Notably, we found no evidence of lateral spreading that could subserve isomorphic filling-in. We did not observe any evidence for increased interactions between the two flicker-tagged inputs, and RIFT responses increased in both central and peripheral ROIs independently. The RIFT result provides an additional argument against lateral interactions. If an oscillating neuron excites another through horizontal connections, each transmission would introduce a phase delay corresponding to the conduction and synaptic times along variable-length axons. The accumulation of such delays across the neuronal population would reduce population-level phase synchrony with the photodiode signal, yielding a lower PLVt. In contrast, PLVt increased during the critical period leading up to filling-in, indicating that the tagged responses remained tightly phase-locked and are therefore unlikely to arise from laterally propagated activity. These results are not consistent with models in which lateral spreading of feedforward excitation across visual cortex serves as the primary substrate of perceptual filling-in in this paradigm. The neuronal-level dynamics of lateral spreading could be investigated through future simulation work based on our findings that may generate hypotheses for further testing. Note that it remains possible that feedback activity from higher-order areas could trigger an isomorphic representation of a different format, but we cannot test this hypothesis in our data. However, this scenario would still imply both that (i) lateral spreading of feedforward excitation does not cause filling-in and (ii) a higher-order mechanism is required.

Furthermore, these findings remain compatible with traditional conceptions of boundary adaptation in early visual cortex. Edge representations in V1 function to inhibit the spread of excitation between neighboring surfaces, and eliminating this inhibition should inevitably cause lateral spreading (*68*, *69*). However, in our study, such inhibition may only be weakened, not abolished, such that V1 boundary representations remain partially intact during filling-in. This possibility is consistent with evidence that V1 activity is not strictly necessary for the percept of filling-in (*10*, *13*). Instead, our findings may reflect the attenuation or loss of a downstream boundary representation that does not itself mediate local inhibitory containment of visual surface excitation. For instance, our findings mirror an observation (*10*) that single neurons in macaque V2 and V3—not V1—gradually increase in firing rate before perceptual filling-in. De Weerd and colleagues (*10*) proposed that this ramping reflects a mechanism in which inhibition adapts over time. This consistency across studies points to a conserved neurophysiological substrate underlying boundary fading.

Our framework also offers a mechanistic account for counterintuitive findings reported in prior filling-in studies. For decades, it has been known that both spatial and feature-based attention can paradoxically facilitate the filling-in of an attended surface (*6*, *70*). With certain stimulus configurations, attending to an object causes it to effectively disappear into the background. This effect was recently investigated (*14*) with electroencephalography (EEG): steady-state visually evoked potentials (SSVEP) (*71*) at low flicker frequencies (<20 Hz) were found to increase before filling-in of a small target surface, coinciding with a decrease in occipital alpha power. Like our RIFT-based observations, these SSVEP increases suggest heightened excitability preceding the perceptual illusion. The authors attributed their findings to attentional enhancement of the target signal—known to increase local excitability and decrease alpha power (*48*)—which in turn facilitated filling-in due to a so far unknown mechanism. Their findings are fully consistent with our view that a shift in E/I balance toward excitation underlies boundary fading. Filling-in can arise as an unintended consequence when attention suppresses local inhibition to amplify stimulus-evoked neural firing. This explanation reconciles the behavioral effects of attention with present and past neurophysiological findings under a single circuit–level mechanism. Both attention and slow sensory adaptation may independently modulate E/I balance, yielding similar outcomes: perceptual filling-in, reduced alpha power, and enhanced SSVEP/RIFT responses.

Our results also concur with the argument in (*14*) that SSVEP amplitude is not a direct marker of conscious visibility. SSVEP/RIFT responses can become dissociated from subjective perceptual experience, remaining robust even as perceptual boundaries fade and surfaces blend. Instead, fluctuations in SSVEP amplitude may reflect changes in the underlying cortical E/I ratio rather than conscious perception per se. This interpretation could offer a unifying physiological mechanism through which diverse cognitive and attentional processes influence SSVEPs (*71*).

Although we found that microsaccades may transiently shift excitation-inhibition balance toward greater inhibition, they were not associated with increased occipital alpha-band activity (which could indicate higher inhibition) or decreased RIFT response (which would reflect lower excitability). This alpha observation aligns with evidence that microsaccades are related to covert attentional shifts (*72*, *73*) and modulate alpha activity based on eye movement direction and the spatial locus of visual attention. Specifically, alpha power increases in the occipital hemisphere ipsilateral to the direction of microsaccades (*74*). This nuanced relationship suggests that alpha activity is not always a blanket marker of inhibition across the visual cortex. Instead, alpha dynamics likely reflect a more complex interaction between spatial attention and oculomotor signals. It remains plausible that such lateralized alpha modulations could influence filling-in, particularly in designs where the stimulus is presented only in only one visual hemifield and microsaccade direction becomes task-relevant. Regarding RIFT phase-locking, within our proposed E/I framework, microsaccades would be expected to reduce RIFT responses by reinstating inhibition in visual cortex. However, microsaccades are known to stimulate the visual system through multiple ways, beginning at the retina level (*21*). Hence, while they may transiently decrease the E/I ratio in certain cortical regions, they could simultaneously increase retinocortical excitation, potentially offsetting any inhibitory effect in the overall RIFT response. This finding concurs with recent reports that small gaze deviations in the microsaccadic amplitude range (<0.75°) do not modulate RIFT amplitude (*75*, *76*). Despite this complexity, we contend that the local balance between excitation and inhibition in boundary-sensitive cortical circuits is the key determinant of whether filling-in occurs. If microsaccades ensure that cortical processing includes sufficient inhibition to support accurate visual perception, then the perceptual transition to filling-in is less likely to take place—even if other sensory responses are enhanced.

Our findings in the motor cortex support the hypothesis that higher-order evidence accumulation contributes to perceptual filling-in and conscious perception in general. Previous electrophysiology studies have implicated alpha- and beta-band activity in motor cortex as markers of perceptual evidence tracking—distinct from motor preparation itself (*31*, *33*, *35*, *36*, *77*, *78*). Complementary theoretical models of conscious perception suggest that sensory contents reach awareness only when corresponding neural evidence accumulators exceed a decision threshold (*79*–*83*). In our data, beta-band activity in motor cortex gradually declined over the course of filling-in trials, with the lowest point preceding perceptual reports. This progressive decrease may reflect a de-accumulation of boundary-related sensory evidence: Activity peaks early, shortly after stimulus onset, and diminishes over time because of adaptation in visual cortex. In contrast, beta activity remained stable during control trials of matched duration, which did not involve perceptual boundary monitoring. This pattern suggests that motor beta activity tracks the adaptation process, perhaps by accumulating only feedforward excitatory signals and ignoring inhibition. This interpretation could explain why motor beta activity begins to decline early in the trial although the visual cortex maintains a stable E/I ratio until just before filling-in. However, this also implies that beta suppression in motor cortex does not capture the full mechanistic structure of boundary adaptation, which depends critically on the evolving balance between excitation and inhibition. Moreover, while microsaccades reliably delay filling-in, they do not reverse the ongoing beta-band decline, further complicating the functional role of this signal. The precise contribution of motor beta activity to perceptual filling-in thus remains unresolved and merits further investigation.

On the other hand, motor high-alpha activity exhibited a distinct temporal profile: It declined only after visual cortical inhibition appeared to have overadapted relative to excitation. We propose that motor high-alpha activity reflects a specific dimension of perceptual evidence—the capacity of the sensory cortex to reliably process task-relevant input, as indexed by the current balance between excitation and inhibition. In this framework, high-alpha activity increases when microsaccades refresh visual input, stabilizing E/I dynamics in visual cortex. It declines before filling-in once adaptation shifts the E/I ratio toward excitation, rendering bottom-up sensory input less reliable. This high-alpha process may receive input from a broader decision-making network including higher-order visual and parietal regions. When motor high-alpha activity reaches a lower threshold—signaling insufficient reliability of visual evidence—this network may generate a higher-order inference about the contents of the visual field. Specifically, it may infer the absence of a boundary and the percept of a uniform surface, instantiating a symbolic representation of visual input (*28*, *29*, *84*). This proposal is related to the concept of criterion, or detection threshold, in perceptual decision-making, which has recently been linked to alpha-band neural activity in sensorimotor regions (*85*).

More broadly, our findings add to a growing body of evidence—spanning perceptual decision-making (see above) and other domains (*32*, *86*–*88*)—that cortical regions traditionally associated with motor control also participate in perceptual and cognitive processes. Notably, we observed that high-alpha activity in motor cortex was left-lateralized during the task, consistent with the use of the right hand for behavioral responses. This suggests that task context—particularly the lateralized motor output—helps determine which cortical areas are recruited for conscious perceptual processing. This idea aligns with the recently proposed Joint Determinant Theory of consciousness (*89*), which argues that the neural substrates of consciousness are not anatomically fixed but instead vary depending on both internal (e.g., attention and intention) and external (e.g., task demands) conditions. Perceptual decision-making studies support this view, showing that neural accumulators are localized to motor-output specific regions: parietal or FEF for saccadic responses, and primary motor cortex for hand movements (*31*, *90*). A limitation of the present study is the lack of counterbalancing between left- and right-hand responses. We expect that using the left hand would shift the lateralized high-alpha signal to the contralateral (right) motor cortex. Future studies should test this prediction directly.

Another important future direction involves studying conscious filling-in in no-report paradigms to assess the neural mechanisms of perception without any overt motor output. Such designs require a reliable neural marker to infer perceptual transitions. We suggest that adaptation-related changes in excitation-inhibition balance may provide a promising candidate. If perceptual filling-in can be reliably tracked on a single-trial basis using increases in RIFT responses or decreases in aperiodic parameters, these measures may provide objective, report-free neural indicators of the filling-in percept. In the absence of overt report, other higher-order areas implicated in perceptual decision-making and monitoring of sensory cortical activity, such as regions of prefrontal or parietal cortex (*91*–*94*), may assume the inferential role attributed here to motor cortex.

Last, we acknowledge several additional limitations of the present study. First, although we interpret our findings within an excitation-inhibition framework, we rely on indirect, albeit well-established, neurophysiological proxies for E/I balance. Direct measurements of synaptic activity in visual cortex would offer more definitive evidence. Furthermore, we do not directly measure activity in neurons representing the boundary between the central and peripheral regions. Our findings in visual cortex, combined with the phenomenology of boundary fading, suggest that boundary adaptation before filling-in involves a reduction in inhibition relative to excitation. Future circuit-level studies will be needed to confirm this interpretation. Second, while our microsaccade analyses enabled direct probing of bottom-up visual dynamics during filling-in, we could only infer the role of higher-order processes such as motor cortex involvement. An analogous probe of motor cortex function—for instance, using alpha-rhythmic transcranial magnetic stimulation (*95*)—could test whether modulating motor alpha activity accelerates or delays filling-in. Third, although we interpret the decline in motor high-alpha activity as a correlate of perceptual filling-in, we cannot definitely rule out motor preparation as a contributing factor. However, two observations weigh against this alternative. In control trials involving a uniformly colored stimulus, motor high-alpha power did not decrease until approximately 400 ms before the button press—consistent with known timelines for motor preparation. In contrast, during filling-in trials, this decrease occurred considerably earlier. Although the illusory nature of filling-in could plausibly lead to hesitation and delayed responses, previous studies have shown that response reaction times following filling-in are typically within standard motor preparation windows (*96*, *97*) and do not exceed 1 s. Moreover, our data show that microsaccades selectively counteract the reduction in motor high-alpha (but not beta) power. Given that microsaccades also induce a delay in filling-in, a more parsimonious explanation is that the reduction in motor high-alpha activity is not a consequence of motor preparation but rather a constituent of the filling-in process.

In summary, this study introduces a comprehensive physiological framework for conscious perceptual transitions during illusory filling-in. We show that subjective reports of filling-in are preceded by electrophysiological signatures of reduced neuronal inhibition relative to excitation in visual cortex, consistent with an imbalance of excitation-inhibition dynamics. Concurrently, motor cortical activity, particularly in the high-alpha band, decreases gradually until the report of filling-in, suggesting that motor-region dynamics are coupled to perceptual decision-making processes. Microsaccades were found to delay the emergence of the filling-in percept while simultaneously counteracting both visual and motor cortical effects. Together, these findings support a hierarchical visuo-motor framework in which lower-order boundary adaptation is accompanied by higher-order cortical dynamics that are temporarily aligned with the perceptual transition to filling-in.

## MATERIALS AND METHODS

### Participants

Thirty participants were recruited through email advertisements published on the University of Birmingham’s Centre for Human Brain Health mailing lists. All participants provided written informed consent and were compensated 15 GBP per hour for their time. The experiment was approved by the Research Ethics Board of the McGill University Faculty of Medicine and Health Sciences (protocol 2021-7130) and the University of Birmingham Ethics Committee (program ERN_18-0226P26A). All participants had normal or corrected-to-normal vision, were right-handed, and were neurologically healthy. One participant’s data were excluded because of a failure to adhere to task instructions, resulting in a final analysis on 29 participants.

### Experimental design

Participants first received written and oral instructions for each task component. A 2-min empty-room MEG noise recording was conducted to inform MEG source mapping. The MEG session comprised a 3-min eyes-open resting state recording, a 1-min flicker localizer, and a 60- to 90-min main task split into seven blocks of 8 to 12 min each. If time permitted, a second 3-min resting state recording was acquired, but resting state data are not reported here.

### Stimuli and behavioral tasks

Stimuli were generated using Psychtoolbox 3 (*98*) and presented using Matlab R2019b (The MathWorks Inc.) with a PROPixx lite projector (VPixx Technologies) that uses a linear gamma function. The radial uniformity illusion stimulus (Fig. 1A) consisted of a central gray circle (referred to as the “center,” 4° visual angle radius,) and a perceptually isoluminant purple background (referred to as the “periphery”) that filled the remainder of a 30.75°-by-17.3° rectangle. The center and periphery smoothly transitioned into each other across a 0.48°-diameter ring surrounding the center. We opted for perceptual isoluminance, as opposed to physical isoluminance, to ensure that the phenomenological experience of filling-in was restricted to color. Colors were derived from an estimation of the isoluminant plane of Derrington-Krauskopf-Lennie (DKL) color space (*99*), with a radius of 0.07 and elevation of 0 relative to a neutral gray baseline [red-green-blue (RGB) 77,77,77]. The stimulus’ color contrast was quantified as the difference in DKL azimuth (hue) values between the center and periphery. We included two possible periphery colors, and half of the trials were classified as “low contrast” (azimuth 285; RGB 32,22,30) while the other half were “high contrast” (azimuth 330; RGB 38,20,30). The center was always assigned a hue of 270° (RGB 26,24,30). We included two contrast levels because higher contrast boundaries typically take longer to fill-in (*4*), and we planned to assess neural correlates of this difficulty effect. Color values were derived from isoluminance calibrations performed in a previous study (*49*), and we further confirmed perceptual isoluminance between the central and both peripheral colors in a pilot test of five participants performing heterochromatic flicker photometry (none of whom were participants in the present study). RGB values were converted from DKL using the Computational Colour Science Toolbox 2e (*100*) and custom MATLAB code.

The primary task consisted of six filling-in blocks and one control block, each comprising 24 trials. The first two participants completed 26 trials per block, but the trial count was subsequently lowered to 24 per block to reduce the overall experiment duration. At the onset of each trial (Fig. 1B), the participants were prompted to press the space bar upon their readiness to begin. The central fixation target subsequently appeared. The participants were instructed to maintain fixation and minimize blinks whenever the fixation cross was displayed on the screen. Following a 3-s interval, the stimulus was displayed behind the fixation cross.

For the filling-in task, the participants were instructed to press a button with their right index finger immediately after they perceived the center and the periphery regions as fully merging into a uniform-colored display. The participants were informed that this was an illusion, with the image on screen not actually changing. In postexperiment debriefing, all participants reported that the final perceived color was always a mixture of the two rather than one color replacing another. They were also instructed to continue maintaining fixation and minimizing blinking after pressing the button, until the fixation cross was no longer displayed. The fixation cross and stimulus disappeared 3 s after button press or after 15 s of stimulus presentation, whichever occurred sooner. After stimulus offset, dynamic colored noise was presented across the display for a duration equivalent to half the time of the stimulus presentation to alleviate retinal fatigue and possible afterimage experiences. The noise was presented atop an inversion of the filling-in stimulus, wherein the central and peripheral hues were each shifted 180° (Fig. 1B, bottom).

The task comprised two additional conditions. Within each filling-in block, two trials were replay catch trials, wherein the center and periphery gradually merged into a uniformly colored stimulus over a 3-s period. These replay trials were designed to simulate the perceptual experience of filling-in and ensure that participants correctly reported filling-in at the intended time. The replay effect commenced randomly between 4 and 6 s after stimulus onset. The 3-s duration for replay, much longer than the transition duration of illusory filling-in, was determined through piloting to minimize afterimages caused by fast contrast decrements (*50*) while ensuring a smooth transition that resembled the illusory filling-in experience. In addition, replay presentation may interact with ongoing visual adaptation mechanisms in unpredictable ways [e.g., (*79*)]. Therefore, after confirming accurate behavior, these trials were not analyzed further.

To control for general effects of visual stimulation and button presses, we included a control condition during the seventh trial block. In these trials, the center and periphery regions were identically colored from stimulus onset (RGB: 29,23,30), thereby eliminating the possibility of perceptual filling-in. Instead, the participants were instructed to press a button whenever the central fixation cross changed color between red and blue, which could occur multiple times within a single trial.

Color changes began randomly between 1 and 6.5 s after stimulus onset, with a 15% likelihood of change on each display frame. The critical color change—the event of interest—always occurred precisely at 6.5 s after stimulus onset, aligning the timing with the typical emergence of the filling-in percept in experimental trials. Neural activity was analyzed in the time window surrounding the first button press that followed this critical change and was compared directly to filling-in trials. Any earlier changes were not analyzed; their sole purpose was to sustain participant engagement and reduce predictability regarding the timing of the upcoming critical change. Each control stimulus remained on screen for 10 s per trial, matching the typical total duration of filling-in trials.

The flicker localizer task used the same center and periphery regions but both colored in gray (RGB 128,128,128). The participants were instructed, as in the control task, to maintain fixation on the central cross for 60 s, minimizing blinks, and to press the button upon the cross’s transition between red and blue. This task was designed to elicit consistent evoked brain responses from the flickering stimulus, as outlined in the following section.

### RIFT presentation

Visual stimuli were projected on a back-projection screen positioned 107 cm in front of the participants. The PROPixx projector transformed 120-Hz graphic inputs into 480-Hz presentations by dividing each 1920 pixel–by–1080 pixel frame into four quadrants of 960 pixel by–540 pixels. These four quadrants were sequentially displayed for 2 ms each. Sinusoidal modulations were applied to the stimuli’s RGB values such that the center area of the stimulus flickered at a frequency of fc = 64 Hz, while the peripheral area flickered at a frequency of fp = 56 Hz. The intensity of the stimuli varied from a value of 0 (black, corresponding to the trough of the sinusoid) to a value of 2 (double perceived intensity, corresponding to the peak of the sinusoid). Because the PROPixx projector has linear gamma, a sinusoidal curve of RGB values resulted in an identical sinusoidal luminance projection. Since the rapid flicker rates exceeded typical flicker fusion thresholds, the participants perceived constant colors corresponding to the sinusoid midpoints. The RIFT method was selected to “tag” neural activity without introducing discernible flicker or entraining endogenous neural oscillations (*40*), which could otherwise disrupt visual processing. RIFT was applied identically to all task conditions.

### Data acquisition

Four head-position indicator coils were attached to monitor head position during MEG recordings. Two of these coils were positioned on the forehead (left and right), and one was positioned below each ear. The three-dimensional locations of these coils, along with those of three fiducial anatomical locations (nasion, left/right tragus-helix junction) and approximately 200 scalp points, were digitized (Polhemus Fastrak) to coregister the MEG recordings with individual anatomical MRI volumes. Oculomotor and cardiac signals were monitored with three bipolar electrode channels: vertical electrooculogram (EOG) from above and below the left eye, horizontal EOG from laterally to both eyes, and electrocardiogram (ECG) from both clavicles. A ground electrode was placed on the left forearm. Participants were seated in an upright position (60°) in an unlit magnetically shielded room (Vacuumschmelze GmbH & Co., Hanau, Germany). They were instructed to rest the back and top of their head against the inner surface of the MEG sensor helmet. Brain activity was recorded with a Neuromag Triux system (Elekta Neuromag, Helsinki, Finland) comprising 204 orthogonal planar gradiometers and 102 magnetometers. Data sampling rate was set to 2000 Hz with an online band-pass filter set between 0.03 and 660 Hz. Two photodiodes were placed at the lower corners of the back-projection screen to precisely record the timing of stimulus flickers concurrently with MEG traces. Head position was measured at the onset of each recording block.

During the flicker localizer and main task conditions, binocular gaze and pupil diameter were continuously monitored at 1000 Hz using an EyeLink 1000 Plus infrared video tracker (SR Research, Ottawa, Canada). A nine-point calibration and validation of the eye tracker were performed at the beginning of each task block. During the main task conditions, if eye gaze deviated more than 3° of visual angle from a central fixation point for at least 67 ms (eight continuous frames), a trial was aborted, and the participant was reminded to maintain eye fixation. During the flicker localizer condition, a gaze deviation of more than 3° triggered a blink of the fixation cross to remind participants to center their gaze. We used a 3° fixation window during acquisition to reduce trial restarts from brief tracking noise or clustered microsaccades. Post hoc inspection revealed that only 1.76% of filling-in trials still contained a large saccade, and excluding these did not alter any main results. Technical difficulties disrupted the recording of eye tracking for two participants, whose data were subsequently excluded from the eye movement analyses (e.g., Fig. 5) presented herein. For all other analyses, these two participants’ data were retained as they do not depend on eye-tracking measures.

For each participant, a T1-weighted brain MRI volume (3 T, Siemens MAGNETOM Prisma, sagittal MP-RAGE sequence, 1 mm–by–1 mm voxels) was either newly acquired after the MEG session or, if available, a scan acquired prior at the same facility, using the same settings, was retrieved. High-resolution cortical surfaces were obtained using Freesurfer’s (*101*) recon-all command (version 7.2.0, default parameters) and subsequently downsampled to 15,000 triangle vertices using Brainstorm. To coregister individual MRI volumes with MEG recordings, the same three fiducial points (nasion, left/right tragus-helix junction) were first identified in the MRI volume. We then applied an iterative closest point rigid-body registration between the digitized scalp points and the triangulated scalp surface (Brainstorm) (*102*) . Four participants declined to participate in the MRI session; for these participants, we used Brainstorm to warp the ICBM152 anatomical template to their respective head shapes inferred from the digitized scalp points collected during the MEG session.

### Microsaccade detection

Microsaccades were defined using a velocity-based algorithm adapted from (*72*, *103*). Briefly, we analyzed the eye-tracking time series to detect ballistic binocular eye movements with an amplitude of 3 arc min to 2° of visual angle lasting at least 8 ms. Time points 150 ms before or after a blink were disregarded. Detected microsaccades followed the expected “main sequence” linear relationship between amplitude and peak velocity (fig. S6A). Microsaccade onsets were imported as event markers for subsequent analyses with the MEG recordings using Brainstorm. In addition, to visualize microsaccade dynamics around filling-in reports (button presses), microsaccade onset events were combined across trials and converted to a microsaccades-per-second rate using a causal windowing function (window length 1001 ms, alpha 1/100) (*104*). These microsaccade rates were converted to percent change from a baseline period defined as (−3.5, −2) seconds relative to button presses. Raw, nonnormalized microsaccade rates in the same time window are shown in fig. S6B.

### MEG preprocessing

MEG preprocessing was performed with Brainstorm, unless otherwise specified, with default parameters and adhering to good-practice guidelines (*105*). Environmental noise was attenuated using signal space separation using MNE-Python (*106*, *107*). The MEG recordings were subsequently notch-filtered at 50 Hz and harmonics (100, 150, and 200 Hz) using a second-order infinite impulse response (IIR) filter to eliminate powerline contamination. The data were then band-pass filtered between 0.05 and 200 Hz using an even-order linear phase finite impulse response (FIR) filter with a 60-dB stopband attenuation. Heartbeats and eye blinks were automatically detected from the ECG and EOG signals, respectively. These events were used to define signal-space projectors separately for gradiometers and magnetometers. The resulting projector topographies were manually inspected to identify those corresponding to stereotypical cardiac and ocular artifacts. Custom projectors were created as necessary when other artifact types were observed in individual power spectrum densities, for instance, highlighting problematic channels. The MEG recordings were subsequently orthogonally projected away from these components. Last, additional transient artifactual segments (associated with, for example, motion, muscle, or SQUID jumps) were manually marked and excluded from further analyses.

The preprocessed data were then downsampled to 500 Hz and epoched around three trial events of interest: stimulus onsets (−1000 to +4500 ms), button presses (−3500 to +1500 ms), and microsaccades (−500 to +1000 ms). All epochs were extended by at least 500 additional milliseconds on both ends to account for the duration of edge effects induced by time-frequency decompositions. These epoch buffer segments were discarded from further analyses. Epochs containing segments with poor quality were rejected. The flicker localizer data were epoched around stimulus onset (−3, 63 s). Individual MEG forward models were derived using the overlapping-sphere approach, and source time series for every epoch were reconstructed using cortically constrained minimum-norm source estimation (*108*) using Brainstorm’s default parameter setting.

### Regions of interest

We defined four ROIs in source space for each participant, corresponding to central and peripheral visual cortex, in both hemispheres. These ROIs were determined functionally, based on responses to the central and peripheral flicker frequencies during the flicker-localizer condition.

To identify responsive cortical sources, we computed the phase-locking value (PLV) (*109*) between each cortical source and photodiode reference time series recorded at 56 Hz (center) and 64 Hz (periphery) using narrow frequency bands (55 to 57 Hz and 63 to 65 Hz). The PLV quantifies the consistency of phase differences between two signals across time, providing a robust index of frequency-specific coordination. Individual cortical PLV maps were smoothed using a 2-mm full width at half maximum (FWHM) Gaussian kernel in Brainstorm. We then retained the 2000 most posterior cortical sources showing the strongest phase-locking, ensuring that the ROIs were spatially restricted to occipital areas. The resulting map was then thresholded to include the top 30% valued sources of each hemisphere. The smoothing and thresholding parameters were determined manually on the data of the first participant and subsequently applied identically to all participants.

Because higher-frequency visual stimuli elicit weaker cortical responses (*110*), the 56- and 64-Hz PLVs could not be directly compared quantitatively. Consequently, we normalized each of the thresholded PLV maps between 0 and 1, with a value of 1 corresponding to the maximum PLV value in each hemisphere. We subtracted these two normalized PLV maps (64 minus 56 Hz) from each other to identify flicker-selective cortical sources. Positively valued sources preferentially responded to the central 64-Hz flicker stimulation, and negatively valued sources preferentially responded to the peripheral 56-Hz flicker stimulation. Last, we delineated all four ROIs—two flicker frequencies in two hemispheres—as clusters of 100 contiguous sources with the highest (for 64 Hz) or lowest (for 56 Hz) flicker selectivity values.

We defined ROIs in the motor cortex from the early M50 cortical response following button presses. We averaged button-press MEG epochs across trials and band-pass filtered the resulting sensor signals between 1 and 80 Hz. To extract the M50, we averaged the absolute value of each cortical time series across 30 to 50 ms following button press. The left motor cortex ROI was defined as a cluster of 100 contiguous sources with the largest resulting M50 values, visually confirmed to overlap with the precentral gyrus. We then symmetrically projected this left motor region onto the right hemisphere to define a homologous control region.

### Phase-locking of cortical time series with photodiode or template sinusoid signals

For the primary task data, we quantified modulations of bottom-up cortical excitability, reflecting the responsiveness of neural circuits to sensory input, using a PLVt between each cortical source time series and the reference photodiode signals that captured the stimulus flickers. PLVt was determined from the Hilbert transform of the time series within a narrow frequency band of interest around each flicker frequency (55 to 57 and 63 to 65 Hz). The magnitude of PLVt reflects the consistency of the cross-trial phase difference between two signals (cortical versus reference) at each time point. The maximum PLVt value of 1 indicates that the phase difference between a photodiode reference signal and a cortical source signal is identical across trials. We used this methodology to also measure PLVt between the cortical time series filtered around flicker intermodulation frequencies (a multiple of the difference between fc and fp): 8 Hz (using a 7- to 9-Hz filter band-pass) and 16 Hz (15- to 17-Hz filter band-pass), with reference sinusoids of 8 and 16 Hz.

### Time-frequency power analysis

Periodograms were computed from the fast Fourier transform (FFT) of each source time series, spanning the frequency range of 1 to 50 Hz, with a sliding window duration of 1000 ms with 100-ms overlapping steps. The resulting FFT coefficients were subsequently squared to estimate the power spectral density (PSD) of cortical signals in a time-resolved manner. These PSD estimates were then averaged across trials.

### Time-resolved estimation of aperiodic spectral power

The power spectrum of neurophysiological activity exhibits a characteristic 1/f shape, with a decrease in signal power with increasing frequency. While spectral peaks at individual frequencies indicate genuine rhythmic (periodic) activity, the background aperiodic component of the spectrum captures nonoscillatory, arrhythmic neural activity (*111*). The characteristics of the aperiodic spectral component reflect the ratio between excitatory and inhibitory neurophysiological activity (*44*). As the E/I ratio increases, either because of increased excitation or decreased inhibition, the slope of the aperiodic spectrum flattens in a log-log scale. This aperiodic flattening can be quantified by decreases in two parameters: the aperiodic exponent (slope) and the offset (overall positive or negative shift).

We used specparam, also referred to as “fitting oscillations and one-over-F” (*43*) to parametrize the resulting PSDs, based on peaks reflecting rhythmic, oscillatory neurophysiological activity, and a 1/f-shaped aperiodic component. This latter was defined in each of the 1000-ms time windows by two parameters: the exponent (slope of the arhythmic component of the PSD) and offset (y intercept).

### Aperiodic correction in visual cortex

We assessed the dynamical power changes of oscillatory cortical activity in the visual cortex by correcting each of the PSDs with the aperiodic activity parameters obtained in the previous aperiodic parametrization step. After log-transforming the PSD values, we subtracted the log-transformed aperiodic spectrum estimated from each time window and converted the resulting corrected values back from log space to obtain a time-resolved measure of oscillatory power unbiased by aperiodic activity at each frequency. We then defined four frequency bands of interest, comprising the alpha band (across 8 to 12 Hz), the beta band (13 to 30 Hz), and the two intermodulation frequencies (fi_1_: 8 Hz, fi_2_: 16 Hz).

### Whole-brain analysis of high-alpha activity

Our exploratory investigations of motor cortex activity suggested that a decrease of high alpha-band (10 to 15 Hz) activity may precede the occurrence of filling-in. To assess the brain-wide distribution of this effect, we Hilbert-transformed all cortical time series within 10 to 15 Hz and subsequently averaged the resulting instantaneous signal amplitude estimates within the time interval −1.25 to −0.75 s immediately preceding button presses. In addition, we investigated the microsaccadic modulation of cortical activity in this frequency band by averaging the Hilbert amplitudes of cortical sources between 0.5 and 0.75 s following microsaccades.

### Statistical analysis

We averaged the PLVt and time-frequency decompositions of the individual source time series from each ROI. Unless otherwise specified, the resulting decompositions were standardized for each ROI in each participant by calculating the magnitude difference (for PLVt) or percent change (for oscillatory power) from the average over a baseline period taken from each epoch: from −1 to −0.5 s for stimulus onset, from −3.5 to −3 s for button press, and from −0.5 to −0.2 s for microsaccades. ROI-based statistical significance was determined using cluster-based permutation tests (5000 permutations, Brainstorm default implementation) on *t* statistics, with a cluster-defining threshold of *P* < 0.05 and a cluster size (sum of contiguous *t* values) threshold of *P* < 0.01. For exploratory time-frequency inference, statistical significance was determined using permutation tests on *t* statistics with FDR correction for multiple comparisons (*q* = 0.01). For whole-brain analyses, we projected individual participant time-frequency decompositions onto the ICBM152 anatomical template, followed by Brainstorm’s spatial smoothing (3-mm FWHM), and then averaged across participants. The resulting whole-brain maps were then thresholded on the basis of uncorrected permuted *t* statistics against zero, with *P* < 0.05 (5000 permutations).

## References

[1] O. Ferrante, U. Gorska-Klimowska, S. Henin, R. Hirschhorn, A. Khalaf, A. Lepauvre, L. Liu, D. Richter, Y. Vidal, N. Bonacchi, T. Brown, P. Sripad, M. Armendariz, K. Bendtz, T. Ghafari, D. Hetenyi, J. Jeschke, C. Kozma, D. R. Mazumder, S. Montenegro, A. Seedat, A. Sharafeldin, S. Yang, S. Baillet, D. J. Chalmers, R. M. Cichy, F. Fallon, T. I. Panagiotaropoulos, H. Blumenfeld, F. P. de Lange, S. Devore, O. Jensen, G. Kreiman, H. Luo, M. Boly, S. Dehaene, C. Koch, G. Tononi, M. Pitts, L. Mudrik, L. Melloni, Adversarial testing of global neuronal workspace and integrated information theories of consciousness. Nature 642, 133–142 (2025). 40307561 10.1038/s41586-025-08888-1 PMC12137136

[2] L. Melloni, L. Mudrik, M. Pitts, C. Koch, Making the hard problem of consciousness easier. Science 372, 911–912 (2021). 34045342 10.1126/science.abj3259

[3] A. K. Seth, T. Bayne, Theories of consciousness. Nat. Rev. Neurosci. 23, 439–452 (2022). 35505255 10.1038/s41583-022-00587-4

[4] M. Levinson, S. Baillet, Perceptual filling-in dispels the veridicality problem of conscious perception research. Conscious. Cogn. 100, 103316 (2022). 35358869 10.1016/j.concog.2022.103316

[5] R. S. Weil, G. Rees, A new taxonomy for perceptual filling-in. Brain Res. Rev. 67, 40–55 (2011). 21059374 10.1016/j.brainresrev.2010.10.004 PMC3119792

[6] L. Lou, Selective peripheral fading: Evidence for inhibitory sensory effect of attention. Perception 28, 519–526 (1999). 10664791 10.1068/p2816

[7] D. ( I. P. V.) Troxler, Ueber das Verschwinden gegebener Gegenstande innerhalb unseres Gesichtskreises. Ophthalmologische Bibliothek 2, 1–119 (1804).

[8] V. S. Ramachandran, R. L. Gregory, Perceptual filling in of artificially induced scotomas in human vision. Nature 350, 699–702 (1991). 2023631 10.1038/350699a0

[9] M. Otten, Y. Pinto, C. L. E. Paffen, A. K. Seth, R. Kanai, The uniformity illusion: Central stimuli can determine peripheral perception. Psychol. Sci. 28, 56–68 (2017). 28078975 10.1177/0956797616672270

[10] P. De Weerd, R. Gattass, R. Desimone, L. G. Ungerleider, Responses of cells in monkey visual cortex during perceptual filling-in of an artificial scotoma. Nature 377, 731–734 (1995). 7477262 10.1038/377731a0

[11] P.-J. Hsieh, P. U. Tse, BOLD signal in both ipsilateral and contralateral retinotopic cortex modulates with perceptual fading. PLOS ONE 5, e9638 (2010). 20300177 10.1371/journal.pone.0009638 PMC2836375

[12] J. D. Mendola, I. P. Conner, S. Sharma, A. Bahekar, S. Lemieux, fMRI measures of perceptual filling-in in the human visual cortex. J. Cogn. Neurosci. 18, 363–375 (2006). 16513002 10.1162/089892906775990624

[13] M. Suárez-Pinilla, A. K. Seth, W. Roseboom, The illusion of uniformity does not depend on the primary visual cortex: Evidence from sensory adaptation. Iperception 9, 204169518800507 (2018). 10.1177/2041669518800728 PMC6166314 30283623

[14] M. J. Davidson, W. Mithen, H. Hogendoorn, J. J. van Boxtel, N. Tsuchiya, The SSVEP tracks attention, not consciousness, during perceptual filling-in. eLife 9, e60031 (2020). 33170121 10.7554/eLife.60031 PMC7682990

[15] R. S. Weil, J. M. Kilner, J. D. Haynes, G. Rees, Neural correlates of perceptual filling-in of an artificial scotoma in humans. Proc. Natl. Acad. Sci. U.S.A. 104, 5211–5216 (2007). 17360383 10.1073/pnas.0609294104 PMC1829288

[16] F. J. J. Clarke, S. J. Belcher, On the localization of troxler’s effect in the visual pathway. Vision Res. 2, 53–68 (1962).

[17] S. Martinez-Conde, S. L. Macknik, X. G. Troncoso, T. A. Dyar, Microsaccades counteract visual fading during fixation. Neuron 49, 297–305 (2006). 16423702 10.1016/j.neuron.2005.11.033

[18] M. B. McCamy, S. L. Macknik, S. Martinez-Conde, Different fixational eye movements mediate the prevention and the reversal of visual fading. J. Physiol. 592, 4381–4394 (2014). 25128571 10.1113/jphysiol.2014.279059 PMC4215783

[19] X. G. Troncoso, S. L. Macknik, S. Martinez-Conde, Microsaccades counteract perceptual filling-in. J. Vis. 8, 15.1–15.9 (2008). 10.1167/8.14.15 19146316

[20] M. Yokota, Y. Yokota, Eye movement inhibits the facilitation of perceptual filling-in. Annu. Int. Conf. IEEE Eng. Med. Biol. Soc. 2010, 6629–6632 (2010). 21096729 10.1109/IEMBS.2010.5627140

[21] S. Martinez-Conde, S. L. Macknik, X. G. Troncoso, D. H. Hubel, Microsaccades: A neurophysiological analysis. Trends Neurosci. 32, 463–475 (2009). 19716186 10.1016/j.tins.2009.05.006

[22] M. V. Sanchez-Vives, L. G. Nowak, D. A. McCormick, Cellular mechanisms of long-lasting adaptation in visual cortical neurons in vitro. J. Neurosci. 20, 4286–4299 (2000). 10818164 10.1523/JNEUROSCI.20-11-04286.2000 PMC6772630

[23] M. V. Sanchez-Vives, L. G. Nowak, D. A. McCormick, Membrane mechanisms underlying contrast adaptation in cat area 17 in vivo. J. Neurosci. 20, 4267–4285 (2000). 10818163 10.1523/JNEUROSCI.20-11-04267.2000 PMC6772627

[24] S. Grossberg, E. Mingolla, Neural dynamics of form perception: Boundary completion, illusory figures, and neon color spreading. Psychol. Rev. 92, 173–211 (1985). 3887450

[25] L. Spillmann, P. De Weerd, “Mechanisms of surface completion: Perceptual filling-in of texture,” in *Filling-In: From Perceptual Completion to Cortical Reorganization*, L. Pessoa, P. De Weerd, Eds. (Oxford Univ. Press, 2003), pp. 81-105. 10.1093/acprof:oso/9780195140132.003.0005.

[26] P. De Weerd, “Perceptual filling-in: More than the eye can see,” in *Progress in Brain Research*, S. Martinez-Conde, S. L. Macknik, L. M. Martinez, J.-M. Alonso, P. U. Tse, Eds. (Elsevier, 2006), vol. 154 of *Visual Perception*, pp. 227–245. http://sciencedirect.com/science/article/pii/S0079612306540129. 10.1016/S0079-6123(06)54012-9 17010714

[27] R. Kanai, D.-A. Wu, F. A. J. Verstraten, S. Shimojo, Discrete color filling beyond luminance gaps along perceptual surfaces. J. Vis. 6, 1380–1395 (2006). 17209741 10.1167/6.12.4

[28] D. C. Dennett, *Consciousness Explained* (Little, Brown and Co, 1991).

[29] J. K. O’Regan, Solving the “real” mysteries of visual perception: The world as an outside memory. J. Psychol. 46, 461–488 (1992). 10.1037/h0084327 1486554

[30] S. M. Fleming, B. Maniscalco, Y. Ko, N. Amendi, T. Ro, H. Lau, Action-specific disruption of perceptual confidence. Psychol. Sci. 26, 89–98 (2015). 25425059 10.1177/0956797614557697 PMC4361353

[31] J. Kubanek, L. H. Snyder, B. W. Brunton, C. D. Brody, G. Schalk, A low-frequency oscillatory neural signal in humans encodes a developing decision variable. Neuroimage 83, 795–808 (2013). 23872495 10.1016/j.neuroimage.2013.06.085 PMC3815962

[32] B. Morillon, S. Baillet, Motor origin of temporal predictions in auditory attention. Proc. Natl. Acad. Sci. U.S.A. 114, E8913–E8921 (2017). 28973923 10.1073/pnas.1705373114 PMC5651745

[33] V. Wyart, V. de Gardelle, J. Scholl, C. Summerfield, Rhythmic fluctuations in evidence accumulation during decision making in the human brain. Neuron 76, 847–858 (2012). 23177968 10.1016/j.neuron.2012.09.015 PMC3975574

[34] D. Goueytes, F. Stockart, A. Robin, L. Gyger, M. Rouy, D. Hoffmann, L. Minotti, P. Kahane, M. Pereira, N. Faivre, Evidence accumulation in the pre-supplementary motor area and insula drives confidence and changes of mind. Nat. Commun. 16, 6998 (2025). 40739086 10.1038/s41467-025-61744-8 PMC12311133

[35] C. A. Devine, C. Gaffney, G. M. Loughnane, S. P. Kelly, R. G. O’Connell, The role of premature evidence accumulation in making difficult perceptual decisions under temporal uncertainty. eLife 8, e48526 (2019). 31774396 10.7554/eLife.48526 PMC6904213

[36] T. H. Donner, M. Siegel, P. Fries, A. K. Engel, Buildup of choice-predictive activity in human motor cortex during perceptual decision making. Curr. Biol. 19, 1581–1585 (2009). 19747828 10.1016/j.cub.2009.07.066

[37] S. Baillet, Magnetoencephalography for brain electrophysiology and imaging. Nat. Neurosci. 20, 327–339 (2017). 28230841 10.1038/nn.4504

[38] N. Seijdel, T. R. Marshall, L. Drijvers, Rapid invisible frequency tagging (RIFT): A promising technique to study neural and cognitive processing using naturalistic paradigms. Cereb. Cortex 33, 1626–1629 (2023). 35452080 10.1093/cercor/bhac160 PMC9977367

[39] A. Zhigalov, J. D. Herring, J. Herpers, T. O. Bergmann, O. Jensen, Probing cortical excitability using rapid frequency tagging. Neuroimage 195, 59–66 (2019). 30930309 10.1016/j.neuroimage.2019.03.056 PMC6547046

[40] K. Duecker, T. P. Gutteling, C. S. Herrmann, O. Jensen, No evidence for entrainment: Endogenous gamma oscillations and rhythmic flicker responses coexist in visual cortex. J. Neurosci. 41, 6684–6698 (2021). 34230106 10.1523/JNEUROSCI.3134-20.2021 PMC8336697

[41] A. Zhigalov, K. Duecker, O. Jensen, The visual cortex produces gamma band echo in response to broadband visual flicker. PLOS Comput. Biol. 17, e1009046 (2021). 34061835 10.1371/journal.pcbi.1009046 PMC8195374

[42] M. Chini, T. Pfeffer, I. Hanganu-Opatz, An increase of inhibition drives the developmental decorrelation of neural activity. eLife 11, e78811 (2022). 35975980 10.7554/eLife.78811 PMC9448324

[43] T. Donoghue, M. Haller, E. J. Peterson, P. Varma, P. Sebastian, R. Gao, T. Noto, A. H. Lara, J. D. Wallis, R. T. Knight, A. Shestyuk, B. Voytek, Parameterizing neural power spectra into periodic and aperiodic components. Nat. Neurosci. 23, 1655–1665 (2020). 33230329 10.1038/s41593-020-00744-x PMC8106550

[44] R. Gao, E. J. Peterson, B. Voytek, Inferring synaptic excitation/inhibition balance from field potentials. Neuroimage 158, 70–78 (2017). 28676297 10.1016/j.neuroimage.2017.06.078

[45] B. F. Händel, T. Haarmeier, O. Jensen, Alpha oscillations correlate with the successful inhibition of unattended stimuli. J. Cogn. Neurosci. 23, 2494–2502 (2010). 20681750 10.1162/jocn.2010.21557

[46] O. Jensen, A. Mazaheri, Shaping functional architecture by oscillatory alpha activity: Gating by inhibition. Front. Hum. Neurosci. 4, 186 (2010). 21119777 10.3389/fnhum.2010.00186 PMC2990626

[47] Y. J. Zhou, L. Iemi, J.-M. Schoffelen, F. P. de Lange, S. Haegens, Alpha oscillations shape sensory representation and perceptual sensitivity. J. Neurosci. 41, 9581–9592 (2021). 34593605 10.1523/JNEUROSCI.1114-21.2021 PMC8612475

[48] A. Zhigalov, O. Jensen, Alpha oscillations do not implement gain control in early visual cortex but rather gating in parieto-occipital regions. Hum. Brain Mapp. 41, 5176–5186 (2020). 32822098 10.1002/hbm.25183 PMC7670647

[49] M. Levinson, C. C. Pack, S. Baillet, Stimulus-dependent delay of perceptual filling-in by microsaccades. J. Vis. 25, 8 (2025). 10.1167/jov.25.8.8 PMC12248979 40622219

[50] J. G. May, K. M. Tsiappoutas, M. B. Flanagan, Disappearance elicited by contrast decrements. Percept. Psychophys. 65, 763–769 (2003). 12956583 10.3758/bf03194812

[51] R. Bachy, Q. Zaidi, Factors governing the speed of color adaptation in foveal versus peripheral vision. J. Opt. Soc. Am. A Opt. Image Sci. Vis. 31, A220–A225 (2014). 24695173 10.1364/JOSAA.31.00A220

[52] S. A. Engel, G. H. Glover, B. A. Wandell, Retinotopic organization in human visual cortex and the spatial precision of functional MRI. Cereb. Cortex 7, 181–192 (1997). 9087826 10.1093/cercor/7.2.181

[53] D. McLelland, P. M. Baker, B. Ahmed, W. Bair, Neuronal responses during and after the presentation of static visual stimuli in macaque primary visual cortex. J. Neurosci. 30, 12619–12631 (2010). 20861368 10.1523/JNEUROSCI.0815-10.2010 PMC3044879

[54] N. Gordon, J. Hohwy, M. J. Davidson, J. J. A. van Boxtel, N. Tsuchiya, From intermodulation components to visual perception and cognition-a review. NeuroImage 199, 480–494 (2019). 31173903 10.1016/j.neuroimage.2019.06.008

[55] L. Drijvers, O. Jensen, E. Spaak, Rapid invisible frequency tagging reveals nonlinear integration of auditory and visual information. Human Brain Mapp. 42, 1138–1152 (2020). 10.1002/hbm.25282 PMC7856646 33206441

[56] E. A. Bock, J. D. Fesi, S. Baillet, J. D. Mendola, Tagged MEG measures binocular rivalry in a cortical network that predicts alternation rate. PLOS ONE 14, e0218529 (2019). 31295259 10.1371/journal.pone.0218529 PMC6622468

[57] C. Wiest, F. Torrecillos, A. Pogosyan, M. Bange, M. Muthuraman, S. Groppa, N. Hulse, H. Hasegawa, K. Ashkan, F. Baig, F. Morgante, E. A. Pereira, N. Mallet, P. J. Magill, P. Brown, A. Sharott, H. Tan, The aperiodic exponent of subthalamic field potentials reflects excitation/inhibition balance in Parkinsonism. eLife 12, e82467 (2023). 36810199 10.7554/eLife.82467 PMC10005762

[58] J. da S. Castanheira, M. Landry, S. M. Fleming, Quantifying rhythmic and arrhythmic components of brain activity. bioRxiv 678322 [Preprint] (2025); 10.1101/2025.09.24.678322.

[59] J. R. Manning, J. Jacobs, I. Fried, M. J. Kahana, Broadband shifts in local field potential power spectra are correlated with single-neuron spiking in humans. J. Neurosci. 29, 13613–13620 (2009). 19864573 10.1523/JNEUROSCI.2041-09.2009 PMC3001247

[60] K. J. Miller, D. Hermes, C. J. Honey, A. O. Hebb, N. F. Ramsey, R. T. Knight, J. G. Ojemann, E. E. Fetz, Human motor cortical activity is selectively phase-entrained on underlying rhythms. PLOS Comput. Biol. 8, e1002655 (2012). 22969416 10.1371/journal.pcbi.1002655 PMC3435268

[61] N. Brake, F. Duc, A. Rokos, F. Arseneau, S. Shahiri, A. Khadra, G. Plourde, A neurophysiological basis for aperiodic EEG and the background spectral trend. Nat. Commun. 15, 1514 (2024). 38374047 10.1038/s41467-024-45922-8 PMC10876973

[62] W. Klimesch, Alpha-band oscillations, attention, and controlled access to stored information. Trends Cogn. Sci. 16, 606–617 (2012). 23141428 10.1016/j.tics.2012.10.007 PMC3507158

[63] S. Meyberg, M. Werkle-Bergner, W. Sommer, O. Dimigen, Microsaccade-related brain potentials signal the focus of visuospatial attention. Neuroimage 104, 79–88 (2015). 25285375 10.1016/j.neuroimage.2014.09.065

[64] M. X. Cohen, *Analyzing Neural Time Series Data: Theory and Practice* (MIT Press, 2014).

[65] M. A. Sommer, R. H. Wurtz, What the brain stem tells the frontal cortex. I. Oculomotor signals sent from superior colliculus to frontal eye field via mediodorsal thalamus. J. Neurophysiol. 91, 1381–1402 (2004). 14573558 10.1152/jn.00738.2003

[66] D. Veniero, J. Gross, S. Morand, F. Duecker, A. T. Sack, G. Thut, Top-down control of visual cortex by the frontal eye fields through oscillatory realignment. Nat. Commun. 12, 1757 (2021). 33741947 10.1038/s41467-021-21979-7 PMC7979788

[67] C. C. Ruff, F. Blankenburg, O. Bjoertomt, S. Bestmann, E. Freeman, J.-D. Haynes, G. Rees, O. Josephs, R. Deichmann, J. Driver, Concurrent TMS-fMRI and psychophysics reveal frontal influences on human retinotopic visual cortex. Curr. Biol. 16, 1479–1488 (2006). 16890523 10.1016/j.cub.2006.06.057

[68] M. W. Self, T. van Kerkoerle, H. Supèr, P. R. Roelfsema, Distinct roles of the cortical layers of area V1 in figure-ground segregation. Curr. Biol. 23, 2121–2129 (2013). 24139742 10.1016/j.cub.2013.09.013

[69] J. Poort, F. Raudies, A. Wannig, V. A. F. Lamme, H. Neumann, P. R. Roelfsema, The role of attention in figure-ground segregation in areas V1 and V4 of the visual cortex. Neuron 75, 143–156 (2012). 22794268 10.1016/j.neuron.2012.04.032

[70] P. De Weerd, E. Smith, P. Greenberg, Effects of selective attention on perceptual filling-in. J. Cogn. Neurosci. 18, 335–347 (2006). 16513000 10.1162/089892906775990561

[71] A. M. Norcia, L. G. Appelbaum, J. M. Ales, B. R. Cottereau, B. Rossion, The steady-state visual evoked potential in vision research: A review. J. Vis. 15, 4 (2015). 10.1167/15.6.4 PMC4581566 26024451

[72] R. Engbert, R. Kliegl, Microsaccades uncover the orientation of covert attention. Vision Res. 43, 1035–1045 (2003). 12676246 10.1016/s0042-6989(03)00084-1

[73] K. Srinivasan, E. Lowet, B. Gomes, R. Desimone, Stimulus representations in visual cortex shaped by spatial attention and microsaccades. Proc. Natl. Acad. Sci. U.S.A. 122, e2420704122 (2025). 40424126 10.1073/pnas.2420704122 PMC12146699

[74] B. Liu, A. C. Nobre, F. van Ede, Microsaccades transiently lateralise EEG alpha activity. Prog. Neurobiol. 224, 102433 (2023). 36907349 10.1016/j.pneurobio.2023.102433 PMC10074474

[75] K. Arora, C. Husta, F. Bouwkamp, N. Seijdel, S. Bai, Q. Han, L. Kenemans, S. Van der Stigchel, S. Gayet, E. Spaak, S. Chota, L. Drijvers, A collaborative guide to rapid invisible frequency tagging (RIFT): Methods, insights, and recommendations. PsyArXiv edshx_v1 [Preprint] (2025); 10.31234/osf.io/edshx_v1. PMC13277780 42326563

[76] K. Arora, S. Gayet, J. L. Kenemans, S. Van der Stigchel, S. Chota, Dissociating external and internal attentional selection. iScience 28, 112282 (2025). 40248115 10.1016/j.isci.2025.112282 PMC12005331

[77] A. D’Andrea, A. Basti, A. Tosoni, R. Guidotti, F. Chella, S. Michelmann, G. L. Romani, V. Pizzella, L. Marzetti, Magnetoencephalographic spectral fingerprints differentiate evidence accumulation from saccadic motor preparation in perceptual decision-making. iScience 25, 105246 (2022). 36274937 10.1016/j.isci.2022.105246 PMC9579494

[78] S. Haegens, V. Nácher, A. Hernández, R. Luna, O. Jensen, R. Romo, Beta oscillations in the monkey sensorimotor network reflect somatosensory decision making. PNAS 108, 10708–10713 (2011). 21670296 10.1073/pnas.1107297108 PMC3127887

[79] S. A. B. Brown, How to get rich from inflation. Conscious. Cogn. 117, 103624 (2024). 38150781 10.1016/j.concog.2023.103624

[80] R. N. Denison, N. Block, J. Samaha, “What do models of visual perception tell us about visual phenomenology?” in *Neuroscience and Philosophy*, F. De Brigard, W. Sinnott-Armstrong, Eds. (MIT Press, 2022); http://ncbi.nlm.nih.gov/books/NBK583719/. 36095112

[81] Y. H. R. Kang, F. H. Petzschner, D. M. Wolpert, M. N. Shadlen, Piercing of consciousness as a threshold-crossing operation. Curr. Biol. 27, 2285–2295.e6 (2017). 28756951 10.1016/j.cub.2017.06.047 PMC5558038

[82] M. Pereira, P. Megevand, M. X. Tan, W. Chang, S. Wang, A. Rezai, M. Seeck, M. Corniola, S. Momjian, F. Bernasconi, O. Blanke, N. Faivre, Evidence accumulation relates to perceptual consciousness and monitoring. Nat. Commun. 12, 3261 (2021). 34059682 10.1038/s41467-021-23540-y PMC8166835

[83] M. Pereira, D. Perrin, N. Faivre, A leaky evidence accumulation process for perceptual experience. Trends Cogn. Sci. 26, 451–461 (2022). 35382993 10.1016/j.tics.2022.03.003

[84] L. Pessoa, E. Thompson, A. Noë, Finding out about filling-in: A guide to perceptual completion for visual science and the philosophy of perception. Behav. Brain Sci. 21, 723–748 (1998). 10191878 10.1017/s0140525x98001757

[85] Y. J. Zhou, M. W. J. van Es, S. Haegens, Distinct alpha networks modulate different aspects of perceptual decision-making. PLOS Biol. 23, e3003461 (2025). 41124628 10.1371/journal.pbio.3003461 PMC12561919

[86] C. Cont, E. Zimmermann, The motor representation of sensory experience. Curr. Biol. 31, 1029–1036.e2 (2021). 33290742 10.1016/j.cub.2020.11.032 PMC7611541

[87] N. Gharesi, L. Luneau, J. F. Kalaska, S. Baillet, Evaluation of abstract rule-based associations in the human premotor cortex during passive observation. bioRxiv 543581 [Preprint] (2023); https://biorxiv.org/content/10.1101/2023.06.06.543581v1.full.pdf.

[88] E. Rassi, Y. Zhang, G. Mendoza, J. C. Méndez, H. Merchant, S. Haegens, Distinct beta frequencies reflect categorical decisions. Nat. Commun. 14, 2923 (2023). 37217510 10.1038/s41467-023-38675-3 PMC10203257

[89] B. J. He, Towards a pluralistic neurobiological understanding of consciousness. Trends Cogn. Sci. 27, 420–432 (2023). 36842851 10.1016/j.tics.2023.02.001 PMC10101889

[90] J. I. Gold, M. N. Shadlen, Representation of a perceptual decision in developing oculomotor commands. Nature 404, 390–394 (2000). 10746726 10.1038/35006062

[91] S. Kornblith, D. Y. Tsao, How thoughts arise from sights: Inferotemporal and prefrontal contributions to vision. Curr. Opin. Neurobiol. 46, 208–218 (2017). 28942219 10.1016/j.conb.2017.08.016

[92] J. Bae, K. Jung, O. James, S. Suzuki, Y. J. Kim, Frontal engagement in perceptual integration under low subjective visibility. Neuroimage 305, 120984 (2025). 39710313 10.1016/j.neuroimage.2024.120984

[93] M. Michel, J. Morales, Minority reports: Consciousness and the prefrontal cortex. Mind Lang. 35, 493–513 (2020).

[94] F. Stockart, R. Msheik, A. Robin, L. Jurkovičová, D. Goueytes, M. Rouy, R. Mareček, D. Hoffmann, L. Mudrik, R. Roman, M. Brázdil, L. Minotti, P. Kahane, M. Pereira, N. Faivre, Cortical evidence accumulation for visual perception occurs irrespective of reports. Nat. Commun. 16, 1–18 (2025). 41006226 10.1038/s41467-025-63255-y PMC12475450

[95] P. Albouy, S. Baillet, R. J. Zatorre, Driving working memory with frequency-tuned noninvasive brain stimulation. Ann. N. Y. Acad. Sci. 1423, 126–137 (2018). 10.1111/nyas.13664 29707781

[96] M. B. McCamy, J. Otero-Millan, S. L. Macknik, Y. Yang, X. G. Troncoso, S. M. Baer, S. M. Crook, S. Martinez-Conde, Microsaccadic efficacy and contribution to foveal and peripheral vision. J. Neurosci. 32, 9194–9204 (2012). 22764228 10.1523/JNEUROSCI.0515-12.2012 PMC6622220

[97] M. J. Davidson, I. L. Graafsma, N. Tsuchiya, J. van Boxtel, A multiple-response frequency-tagging paradigm measures graded changes in consciousness during perceptual filling-in. Neurosci. Conscious 2020, niaa002 (2020). 32296545 10.1093/nc/niaa002 PMC7151726

[98] D. H. Brainard, The psychophysics toolbox. Spat. Vis. 10, 433–436 (1997). 9176952

[99] A. M. Derrington, J. Krauskopf, P. Lennie, Chromatic mechanisms in lateral geniculate nucleus of macaque. J. Physiol. 357, 241–265 (1984). 6512691 10.1113/jphysiol.1984.sp015499 PMC1193257

[100] S. Westland, Computational Colour Science using MATLAB 2e, (2021); https://mathworks.com/matlabcentral/fileexchange/40640-computational-colour-science-using-matlab-2e.

[101] B. Fischl, FreeSurfer. Neuroimage 62, 774–781 (2012). 22248573 10.1016/j.neuroimage.2012.01.021 PMC3685476

[102] F. Tadel, S. Baillet, J. C. Mosher, D. Pantazis, R. M. Leahy, Brainstorm: A user-friendly application for MEG/EEG analysis. Comput. Intell. Neurosci. 2011, e879716 (2011). 10.1155/2011/879716 PMC3090754 21584256

[103] S. Martinez-Conde, S. L. Macknik, D. H. Hubel, Microsaccadic eye movements and firing of single cells in the striate cortex of macaque monkeys. Nat. Neurosci. 3, 251–258 (2000). 10700257 10.1038/72961

[104] R. Engbert, “Microsaccades: A microcosm for research on oculomotor control, attention, and visual perception,” in *Progress in Brain Research*, S. Martinez-Conde, S. L. Macknik, L. M. Martinez, J.-M. Alonso, P. U. Tse, Eds. (Elsevier, 2006), vol. 154, pp. 177–192. 10.1016/S0079-6123(06)54009-9 17010710

[105] J. Gross, S. Baillet, G. R. Barnes, R. N. Henson, A. Hillebrand, O. Jensen, K. Jerbi, V. Litvak, B. Maess, R. Oostenveld, L. Parkkonen, J. R. Taylor, V. van Wassenhove, M. Wibral, J.-M. Schoffelen, Good practice for conducting and reporting MEG research. Neuroimage 65, 349–363 (2013). 23046981 10.1016/j.neuroimage.2012.10.001 PMC3925794

[106] A. Gramfort, M. Luessi, E. Larson, D. Engemann, D. Strohmeier, C. Brodbeck, R. Goj, M. Jas, T. Brooks, L. Parkkonen, M. Hämäläinen, MEG and EEG data analysis with MNE-python. Front. Neurosci. 7, (2013). 10.3389/fnins.2013.00267 PMC3872725 24431986

[107] S. Taulu, M. Kajola, Presentation of electromagnetic multichannel data: The signal space separation method. J. Appl. Phys. 97, 124905 (2005).

[108] M. S. Hämäläinen, R. J. Ilmoniemi, Interpreting magnetic fields of the brain: Minimum norm estimates. Med. Biol. Eng. Comput. 32, 35–42 (1994). 8182960 10.1007/BF02512476

[109] J. Lachaux, E. Rodriguez, J. Martinerie, F. J. Varela, Measuring phase synchrony in brain signals. Hum. Brain Mapp. 8, 194–208 (1999). 10619414 10.1002/(SICI)1097-0193(1999)8:4<194::AID-HBM4>3.0.CO;2-C PMC6873296

[110] T. Minarik, B. Berger, O. Jensen, Optimal parameters for rapid (invisible) frequency tagging using MEG. Neuroimage 281, 120389 (2023). 37751812 10.1016/j.neuroimage.2023.120389 PMC10577447

[111] B. J. He, Scale-free brain activity: Past, present, and future. Trends Cogn. Sci. 18, 480–487 (2014). 24788139 10.1016/j.tics.2014.04.003 PMC4149861

