# Supplementary material for: Hierarchical brain dynamics supporting visual perceptual transitions
Source: Sci Adv. 2026 May 8;12(19):eaea3919. doi: 10.1126/sciadv.aea3919 (PMC13155359; doi:10.1126/sciadv.aea3919)
Supplement: Supplementary file 1 — Figs. S1 to S6 [file sciadv.aea3919_sm.pdf]

Science Advances
AAAS

Supplementary Materials for

Hierarchical brain dynamics supporting visual perceptual transitions

Max Levinson *et al.*

Corresponding author: Max Levinson, max.levinson@mail.mcgill.ca; Sylvain Baillet, sylvain.baillet@mcgill.ca

Sci. Adv. **12**, eaea3919 (2026)
DOI: 10.1126/sciadv.aea3919

This PDF file includes:

Figs. S1 to S6

Fig. S1.

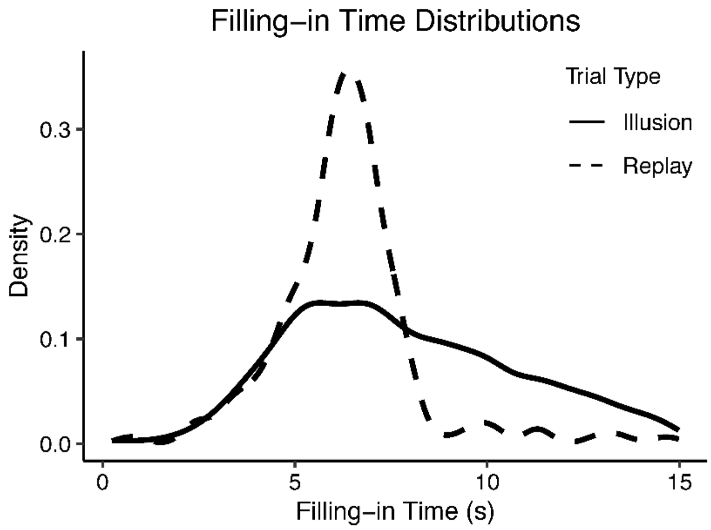

Fig. S1. Distribution of FTs across different trial types. Replay trial FTs (dashed line) clustered around the simulated effect (6–8 seconds). Main trial FTs (solid line) were more broadly distributed, reflecting subjective variability in the filling-in illusion onset.

Fig. S2.

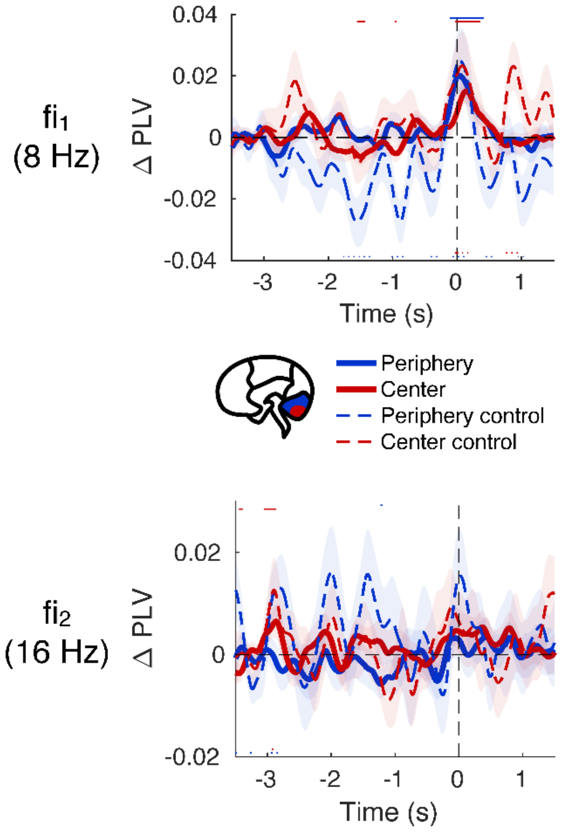

Fig. S2. Intermodulation Phase-Locking Aligned to Filling-in Reports.
Phase-locking values (PLV) of cortical responses to intermodulation reference frequencies (8 Hz and 16 Hz) exhibited stable temporal profiles prior to participants' filling-in button presses. No systematic change in phase-locking was observed preceding subjective filling-in reports.

Fig. S3.

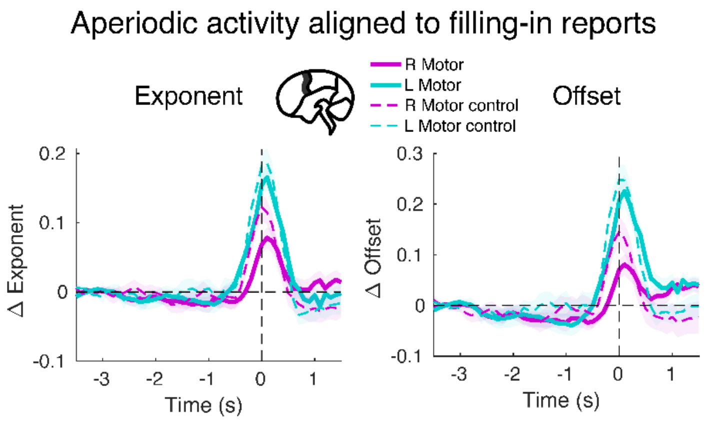

Fig. S3. Aperiodic exponent and offset parameters in motor cortex, aligned to filling-in reports. Both aperiodic parameters increased at the time of the button press.

Fig. S4.

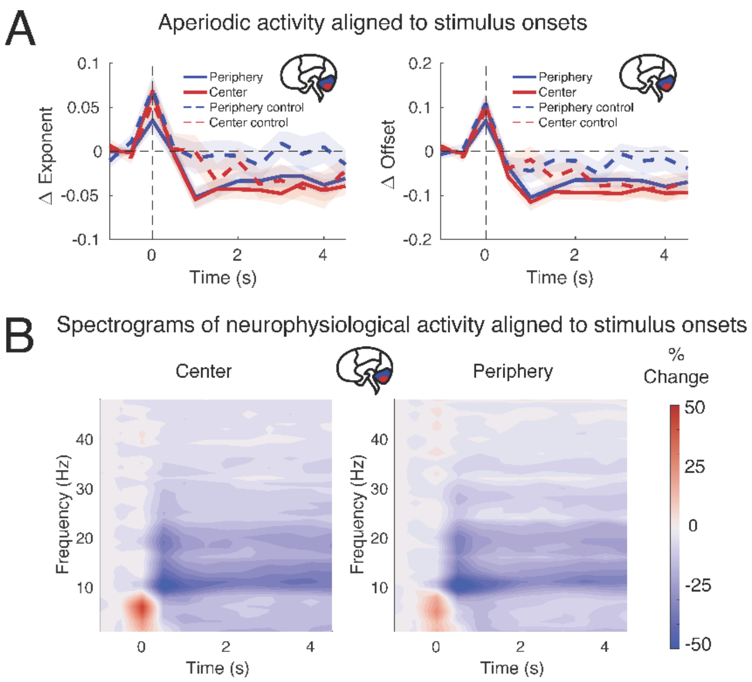

Fig. S4. Neurophysiological activity in the visual cortex, aligned to stimulus onset (0 s).
(A) Aperiodic exponent (left) and offset (right).
(B) Time-frequency decomposition.

Fig. S5.

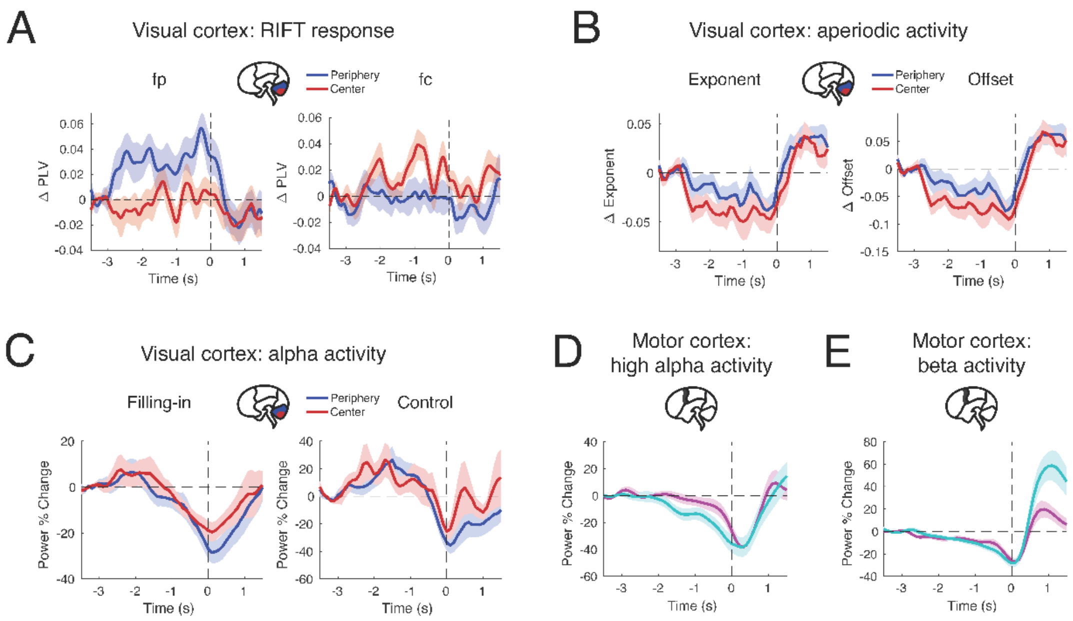

Fig. S5. Cortical Responses Aligned to Button Presses in Trials without Microsaccades.
(A) RIFT cortical responses in visual cortex.
(B) Aperiodic exponent and offset parameters in visual cortex.
(C) Aperiodic-corrected alpha-band (8–12 Hz) oscillatory power in visual cortex.
(D) High-alpha-band (10–15 Hz) oscillatory power in motor cortex.
(E) Beta-band (15–30 Hz) oscillatory power in motor cortex.
All panels show cortical dynamics preceding subjective reports of filling-in, restricted exclusively to trials without detectable microsaccades.

Fig. S6.

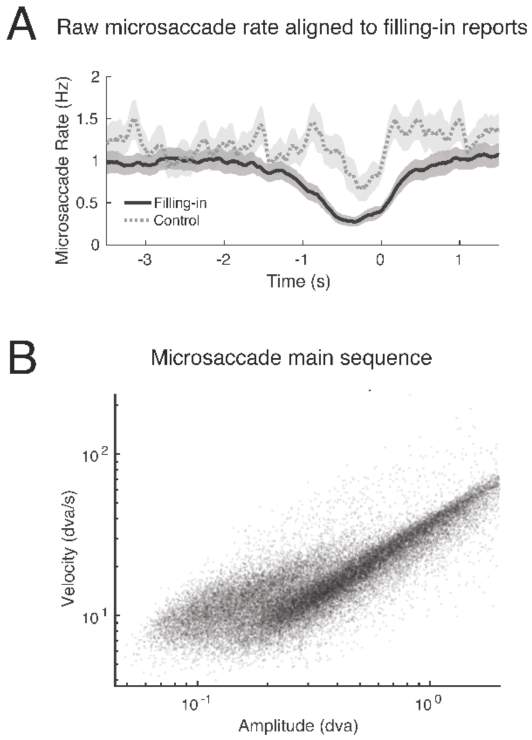

Fig. S6. Microsaccade statistics.
(A) Average raw microsaccade rates aligned to the button press reporting perceptual filling-in.
(B) Main sequence demonstrating a linear relationship between microsaccade amplitude and peak velocity.
